# The intersection of community engagement and team science research: A scoping review

**DOI:** 10.1017/cts.2024.644

**Published:** 2024-10-28

**Authors:** Sarah D. Hohl, Erin Abu-Rish Blakeney, Lori Carter-Edwards, Magaly Ramirez, Sarah Towner Wright, Brenda K. Zierler, Dillon van Rensburg, Teresa Jewell, Linda K. Ko

**Affiliations:** 1 Department of Health Systems and Population Health, University of Washington School of Public Health, Seattle, WA, USA; 2 Department of Family Medicine and Community Health, University of Madison-Wisconsin, Madison, WI, USA; 3 Department of Biobehavioral Nursing and Health Informatics, University of Washington School of Nursing, Seattle, WA, USA; 4 Institute of Translational Health Sciences, University of Washington, Seattle, WA, USA; 5 Department of Health Systems Science, Kaiser Permanente Bernard J. Tyson School of Medicine, Pasadena, CA, USA; 6 Public Health Leadership Program, Gillings School of Global Public Health, and the NC Translational and Clinical Sciences Institute, University of North Carolina at Chapel Hill, Chapel Hill, NC, USA; 7 Health Sciences Library, University of North Carolina at Chapel Hill, Chapel Hill, NC, USA; 8 Health Sciences Library, University of Washington, Seattle, WA, USA

**Keywords:** Community engagement, team science, scoping review, health equity, collaboration

## Abstract

**Introduction:**

Integrating community expertise into scientific teams and research endeavors can holistically address complex health challenges and grand societal problems. An in-depth understanding of the integration of team science and community engagement principles is needed. The purpose of this scoping review was to identify how and where team science and community engagement approaches are being used simultaneously in research.

**Methods:**

We followed Levac’s enhancement of Arksey and O’Malley’s Scoping Review Framework and systematically searched PubMed, CINAHL, Scopus, ERIC, and Embase for team science and community engagement terms through January 2024.

**Results:**

Sixty-seven articles were reviewed. Publications describing integrated team science and community-engaged research have increased exponentially since 2004. Over half were conducted outside of the U.S., utilized qualitative methods, included community-researcher co-development of research question and study design, and described team partnership goals, roles, and management. Fewer studies evaluated partnership, built community capacity, described financial compensation to communities, or described team dynamics facilitation.

**Conclusion:**

As researchers continue to integrate community engagement and team science, common criteria and strategies for integrating the approaches are needed. We provide 19 recommendations for research teams, research institutions, journals, and funding bodies in service of advancing the science and practice of this integration.

## Introduction

Addressing complex societal problems such as health inequality, poverty, and climate and environmental change requires collaboration among teams representing multiple disciplines, sectors, and expertise. As such, funding agencies, research and clinical teams and institutions, and community groups alike are prioritizing research and practices that focus on collaboratively and holistically addressing multiple dimensions of these wicked challenges [1–4]. This phenomenon is evidenced by two intersecting trends. First, investments have grown in research on the relatively nascent field of team science [5–7], – that is, collaborative efforts that integrate strengths of individuals with diverse expertise to address scientific challenges [8], as well as in the emerging field of the Science of Team Science – empirical inquiry of the processes by which scientific teams conduct research [7]. Second, there is increasing engagement across communities, scientific teams, and healthcare systems to cooperatively, equitably, and more effectively investigate and intervene upon health and its determinants [9–13].

With the emphasis on promoting health equity through improved approaches to translational research across the research continuum (i.e., conceptualization, design, implementation, dissemination), the ability to assemble diverse groups to comprehensively answer specific research questions and solve complex problems is even more critical. Team science has historically focused on convening health professionals (e.g., researchers, clinicians, program staff) to conduct interdisciplinary research, and community engagement has historically focused on community inclusion to pragmatically address the needs of a population. Yet, there is an overlap in potential for and requirements of collaboration between, and integration of, these two fields. For instance, Clinical and Translational Science Awardees (CTSAs), funded by the National Institutes of Health National Center for Advancing Translational Sciences (NCATS), are explicitly required to include both team science and community engagement as part of their program to increase translational research efficiency and effectiveness. As diverse teams within and beyond the CTSAs aim to both advance community engagement and team science research and address complex societal challenges, it is important to understand the distinctions, overlap, and complementarity of these two fields. This knowledge can increase the potential for team science to advance community-engaged research and vice versa.

Recent reviews of the community engagement literature have investigated the intersection of community engagement and community-engaged scholarship [2], measures of success [14,15], and its utilization in specific disciplines [16,17], for specific populations [18–21], and to prevent and manage specific diseases [22,23], The few existing reviews of the team science literature have primarily focused on aspects of and influences on collaboration in scientific and interdisciplinary teams [6,24–26], Some exploratory work into synergies of these two areas has occurred, in which investigators provide recommendations [27], competencies [28], and a framework [29,30] for community engagement with science teams. However, this is the first review of which we are aware that investigates the intersection of community engagement and team science.

Integrated community engagement and team science research approaches are both critical and novel, with few tools existing that describe or guide this practice. An in-depth understanding of the science and practice of this integration is needed to guide future researchers, clinicians, policymakers, funders, the public health workforce, and communities to thoughtfully solve complex problems that require partnership. This exploration requires a thorough review of the scientific literature to understand their language of collaboration and produce a summary of the integration of the fields and directions for future interdisciplinary, transdisciplinary, and translational research within and beyond the CTSAs. The purpose of this scoping review was to systematically examine how community engagement and team science intersect in empirical studies. We sought to answer the question: *How do team science research and community engagement research jointly approach research collaboration?* Our aims were to (1) describe the nature and scope of team collaboration in the context of community engagement research as described in empirical studies; (2) articulate the purpose and design of published studies utilizing team science and community engaged research; and (3) describe the overlap of the characteristics of community-engaged and team science research. These aims align with the goals of scoping reviews to identify types of evidence, examine how research is conducted, and identify key characteristics related to a concept [31] (i.e., integration of team science and community engagement).

## Materials and methods

To address the aims of our study while simultaneously achieving the goals of scoping reviews stated above, we followed Levac’s enhancement [32] of methodological framework for scoping reviews [33]. Arksey and O’Malley’s framework includes five phases to (1) identify the research question; (2) identify relevant studies; (3) select studies); (4) chart the data; (5) summarize results [33]. We selected Levac’s scoping review methodology given the addition of a sixth phase--consult external experts. This addition was designed specifically to advance health research by enhancing the application and relevance of scoping studies [32]. In this scoping review, we engaged individuals outside of the scoping review team who represent academic institutions and have expertise in one or both subject areas, as well as community members who collaborate on scientific teams.

### Phase 1. Identifying the research question

To conduct the scoping review, we assembled an interdisciplinary team of nine investigators and librarians from four institutions. Of these, three had expertise in team science, five had expertise in community-engaged research, and one had expertise in both areas of inquiry. The team met approximately twice monthly over 18 months and as needed after studies were selected. In the first meeting, the team agreed upon working definitions to guide this work. The impetus for this review was, in part, driven by the NCATS requirement to utilize both community engagement and team science approaches. Thus, the team relied upon the CTSA Community Engagement Task Force definition of *community engagement*: “a continuum of community involvement,” and the process of “working collaboratively with and through groups of people affiliated by geographic proximity, special interest, or similar situations to address issues affecting the wellbeing of those people [34–36].” We used Vogel et al.’s definition of *team science*: “a collaborative effort to address a scientific challenge that leverages the strengths and expertise of professionals trained in different fields,” where “team members with training and expertise in different fields work together to combine or integrate their perspectives in a single research endeavor [8].” We then collaboratively developed the following research question:

1. How do teams conducting community-engaged research and team science research jointly approach research collaboration?

### Phase 2. Identifying relevant studies

In collaboration with the full study team, the search strategy was designed and implemented by a health sciences librarian (STW) with the intention to search the health and life science literature. Databases searched included: PubMed, Cumulative Index to Nursing and Allied Health Literature via EBSCO, EMBASE via Elsevier, ERIC via EBSCO, and Scopus. Although the search was not restricted by language, articles that did not have an English translation available were excluded. All database results were collected from the inception of the database through January 2024. Search terms were used to retrieve articles addressing the two main concepts of the search strategy: (1) community engagement and (2) team science (Appendix 1). The search was conducted in PubMed using keyword and MeSH combinations. Results from all databases were exported to EndNote. All 1271 references retrieved were uploaded to Covidence systematic review software (https://www.covidence.org), a web-based tool designed to facilitate the abstraction and review process; 280 duplicates were removed. Titles and abstracts of 991 unique citations were screened.

To develop inclusion and exclusion criteria for each area of inquiry, we determined that a study could be included if it described collaboration between at least one research team and at least one community group. We recognized that our team and the scoping review required a clear conceptualization of “community group.” To facilitate a shared understanding of *community group*, the team discussed and agreed upon a definition and examples. We conceptualized *community group* as an entity comprising individuals with a shared identity, a collective interest, and/or one working towards a common purpose *and* not affiliated with an academic or research institution. Examples of community groups included those with a shared identity (e.g., racial or ethnic identity, health condition), community-based organizations, tribal communities, and regional, state, national, or global groups or organizations. To identify characteristics of community engagement and team science, we generated a list of characteristics of each based on: (1) a brief review of characteristics described in the community-based participatory research and community engagement literature [2,4,37–39] and team science literature [40–43]; (2) expertise within our team; and (3) feedback from experts in the field on the list of characteristics of each approach. The final list included 16 community engagement and 12 team science characteristics (Table 1).

**Table 1.** Community engagement and team science characteristics

Community engagement characteristics		
Characteristic		Description
1	Reciprocity	Collaboration grounded in bidirectional exchange, including seeking, recognizing, respecting, and incorporating the knowledge, perspectives, and resources that each partner brings to a collaboration
2	Asset-based approach	Community partners, strengths, skills, and knowledge are respected and incorporated
3	Co-defining the issue	Collaborators work together to define the issue, problem, solution and/or measures of success
4	Addressing community priority	Research study focuses on a need or priority identified by the community
5	Study co-design	Collaborators work together to identify the research question and/or data collection strategy to meet the community priority
6	Instrument co-design	Community participates in design of data collection instruments
7	Study implementation	Community participated in study implementation, such as in data collection
8	Data analysis	Community participated data analysis, interpretation and/or decision-making
9	Coauthors	Community members included as authors on dissemination of findings, e.g., publication, conference presentation, report
10	Communicating study findings	The study team reported the study findings back to the community.
11	Policy implications	Policy or practical implications of the research that will benefit the community
12	Sustainability	Considers long term project maintenance at the outset of the co-design and throughout the research process
13	Community member compensation	Community members are paid members of the study team
14	Advisory boards	Community members are part of advisory boards or councils for the grant or study
15	Ongoing commitment to community	Ongoing commitment to relationships and projects with community members beyond any single funded project
16	Building community capacity	Focus on building community skills that are transferrable to the project and/or priority beyond the current project
Team science characteristics		
1	Organizational context	Organizational context of team research, such as external support, education, and/or rewards
2	Team leadership	Cognitive, motivational, affective, and coordination processes associated with influencing organizational team performance
3	Team partnership goals	The purpose of convening the team and what the team hopes to achieve through collaboration
4	Team member roles	Team members’ functions and responsibilities in the collaboration
5	Leveraging expertise	How roles are needed to leverage strengths and expertise of people with different roles, from different disciplines or sectors. This includes how teams collaborate to achieve breakthroughs unattainable by individual or additive effort
6	Team processes	Team processes developed or utilized to coordinate their work
7	Interpersonal team effectiveness	How each member feels, behaves, and works together
8	Facilitating team affect	Activities collaborators conduct to facilitate an effective team dynamic, such as emotional bonds between team members that are grounded in expressions of genuine care and concern for the welfare of others including empathy and affiliation
9	Communication	Practices and skills for collaborators to exchange and integrate knowledge and expertise through interpersonal, relational, organizational, and pedagogical means
10	Team research management	Specific actions taken to organize, plan, and execute components of the research including integrating efforts of community and academic partners
11	Material resources	Tangible resources, such as infrastructure, that help team members complete tasks efficiently
12	Collaborative problem-solving	Cognitive and social skills allowing teams to integrate group achievements with team members’ idiosyncratic knowledge

Characteristics were developed based on (1) a brief review of characteristics described in the community-based participatory research and community engagement literature [2,4,37–39] and team science literature [40–43]; (2) expertise within our team; and (3) feedback from experts in the field on the list of characteristics of each approach.

### Phase 3. Selecting studies

All titles and abstracts were screened by one community engagement researcher (SH, LK, or MR) and one team science researcher (EB, SH, or BZ) using specific inclusion and exclusion criteria. A study was included if it:

1. Was a data-based paper that included primary or secondary data analysis;
2. Was published in a peer-reviewed journal;
3. Described collaboration between at least one group representative of communities and at least one research team;
4. Described at least 2 characteristics of community engagement (Table 1);
5. Described at least 2 characteristics of team science (Table 1).

A study was excluded if it:

1. Was not a research paper or study;
2. Described an evaluation of a training program or organization;
3. Did not address a minimum of two team science criteria and a minimum of two community engagement characteristics;
4. Was not available in English; or
5. Did not have full text available.

**Figure 1.**
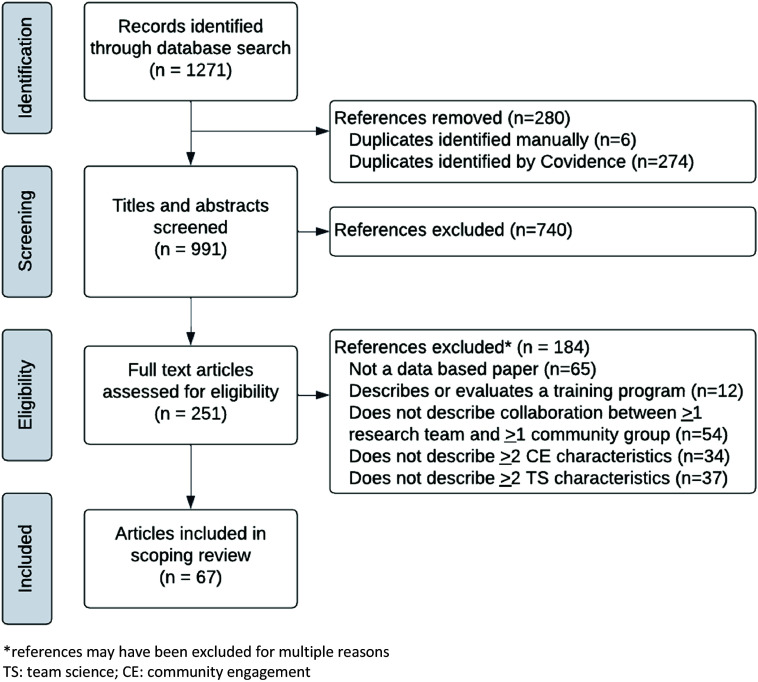
Preferred Reporting Items for Systematic Reviews and Meta-Analyses Extension for Scoping Reviews adapted flow diagram for January 2023 scoping review of team science and community engagement research.

Following title and abstract review, one team science researcher (EB, SH, TJ, BZ) and one community engagement researcher (SH, LK, MR, DV) reviewed each full-text article to confirm that selected articles met the inclusion criteria. We adapted a Preferred Reporting Items for Systematic Reviews and Meta-Analyses-Scoping Review flow diagram (Figure 1) [44]. Disagreements were discussed and resolved by consensus during team meetings.

### Phase 4. Charting the data

Seven team members collaboratively agreed upon variables to include in a data abstraction instrument. The instrument included variables across three sections: (1) study contextual characteristics (e.g., publication year, journal, authors); (2) Scholarly origin, frameworks, theories, and outcomes; (3) Community engagement and team science characteristics. The full instrument is available in Appendix 2. Each team member independently abstracted data from 3 to 5 articles to ensure the abstraction approach was appropriate and that variables were consistent with the purpose of the review. Both the pilot and final instrument utilized REDCap electronic data capture tools [45] to abstract article characteristics. To address potential bias and abstraction quality, 13 (19%) articles were abstracted by two team members [46].

### Phase 5. Summarizing the results

The lead author compared data across abstractors and consulted with two team members in rare instances when conflicts arose. Data from REDCap were uploaded into SPSS (Version 28) statistical software to calculate descriptive statistics. To better understand the overlap of specific characteristics of community engagement and team science, we assessed the strength of associations between community engagement and team science characteristics described across studies using chi-square tests and a significance level of 0.05.

### Phase 6. Consulting external experts

Our team consulted both team science and community engagement experts in two phases of the scoping review. First, to identify and define characteristics of team science and community engagement as part of Phase 1, we sent a list of characteristics and definitions identified in the literature and by team members to colleagues in our team science and community engagement research networks and requested their feedback. We incorporated that feedback into the final list of characteristics and definitions (Table 1). Second, in February 2024, the lead author presented preliminary scoping review results and facilitated a discussion among the CTSA Collaboration and Engagement Enterprise Committee [47] to gain perspectives about translating findings into actionable recommendations. The committee comprises representatives of more than 60 academic/medical institutions, their community partners, and funding agencies who are affiliated with the CTSAs. While community partners from all CTSAs are invited to join the Enterprise Committee, the attendance is largely from academic/medical institutions. The overall vision of the committee is to foster collaboration with community partners and the community that they serve through active engagement and promotion of team science. Two members of the scoping review team were present at the meeting and took notes on participant comments. Comments were discussed with the full scoping review team and integrated into the manuscript discussion and recommendations.

Upon completion of the scoping review methodology, the scoping review team discussed the results during multiple team meetings. We collaboratively developed recommendations based on the scoping review findings and suggestions from CTSA Collaboration and Engagement Enterprise Committee members. To support the development of actionable recommendations, we determined specific audience(s) for each recommendation.

## Results

**Figure 2.**
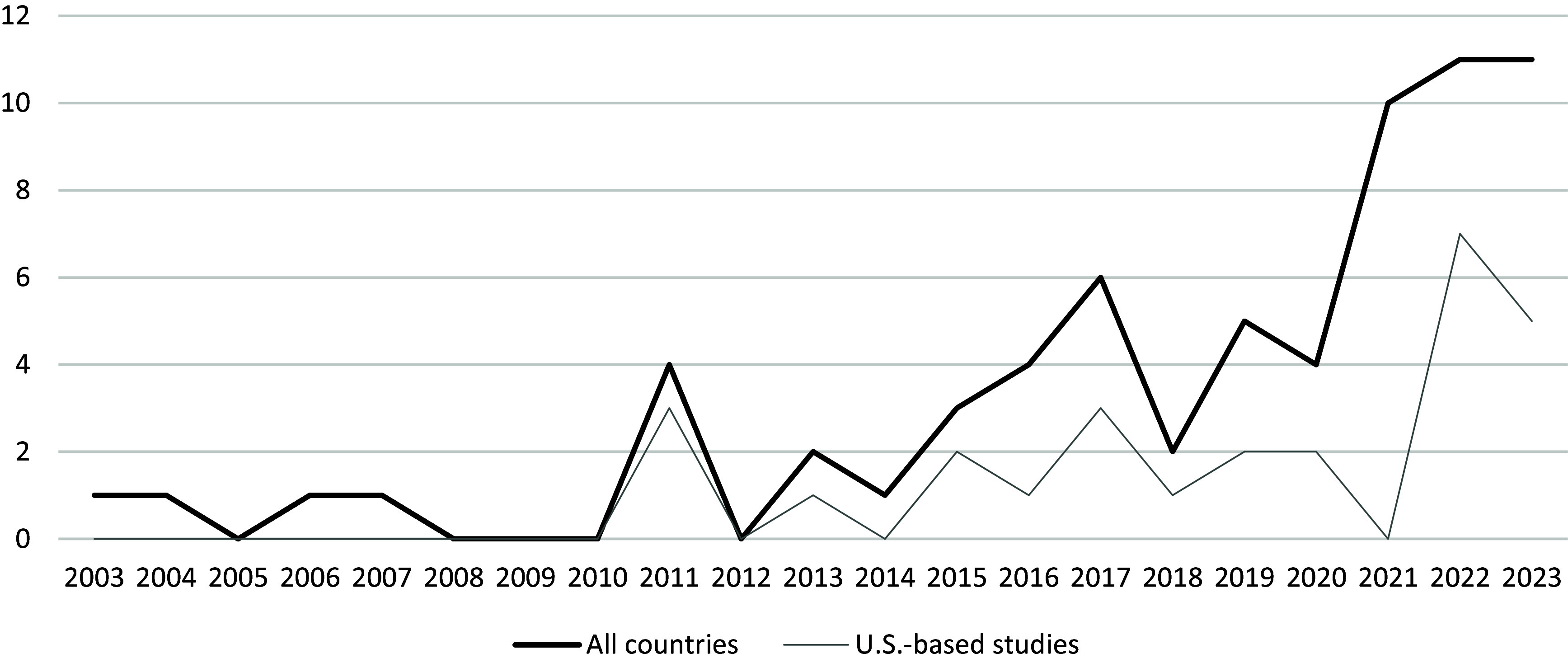
Team science and community engagement publications, 2003–2023.

A total of 1271 unique articles were identified using the initial search strategy (Figure 1). After completing the title and abstract screening, full text of 251 articles was assessed for eligibility, and 67 articles from 54 unique journals were included in the scoping review (Table 2). Figure 1 illustrates exclusion reasons. The time frame for this search was not limited as we intended to provide an overview of all empirical studies utilizing both team science and community-engaged research. Both U.S.-based and those based outside of the U.S. were published with increasing, but not linear, frequency from 2004 to 2023 (Figure 2). No studies had been published in 2024 at the time the search was conducted on January 19, 2024.

**Table 2.** Manuscripts included in review

Authors	Title	Year	DOI	Journal
Alamo-Hernández, U., Espinosa-García, A. C., Rangel-Flores, H., Farías, P., Hernández-Bonilla, D., Cortez-Lugo, M., … & Riojas–Rodríguez, H.	Environmental Health Promotion of a Contaminated Site in Mexico	2019	10.1007/s10393-019-01407-5	Ecohealth
Albert, A.; Islam, S.; Haklay, M.; McEachan, R. R. C.	Nothing about us without us: A co-production strategy for communities, researchers and stakeholders to identify ways of improving health and reducing inequalities	2023	10.1111/hex.13709	Health Expectations
Asojo, A.; Vo, H.; Fisher, T.; Singh, V.	Shaping health and well-being in a COVID era: the role of design	2022	10.1108/ARCH-01-2022-0019	International Journal of Architectural Research: Archnet
Ben, K.; Pozzoboni, K. M.	Student interpretations of a school closure: Implications for student voice in equity-based school reform	2011		Teachers College Record
Berger-Gonzalez, M., Stauffacher, M., Zinsstag, J., Edwards, P., & Kr FC;tli, P.	Transdisciplinary Research on Cancer-Healing Systems Between Biomedicine and the Maya of Guatemala: A Tool for Reciprocal Reflexivity in a Multi-Epistemological Setting	2016	10.1177/1049732315617478	Qual Health Res
Berger-Gonzalez, M.; Scotti, F.; Garcia, A. I.; Hesketh, A.; Hitziger, M.; Thompson, I.; Heinrich, M.	Green Health in Guatemala: How can we build mutual trust and partnerships to develop an evidence-base for local medicines and realize their potential?	2022	10.1139/cjb-2021-0070	Botany
Brewer, L. C.; Hayes, S. N.; Caron, A. R.; Derby, D. A.; Breutzman, N. S.; Wicks, A.; Raman, J.; Smith, C. M.; Schaepe, K. S.; Sheets, R. E.; Jenkins, S. M.; Lackore, K. A.; Johnson, J.; Jones, C.; Breitkopf, C. R.; Cooper, L. A.; Patten, C. A.	Promoting cardiovascular health and wellness among African-Americans: Community participatory approach to design an innovative mobile-health intervention	2019	10.1371/journal.pone.0218724	PLoS One
Bueno, I., Moreno-Calles, A. I., & Merçon, J.	Yeknemilis: Social Learning and Intercultural Transdisciplinary Collaboration for Sustainable Life	2023	10.3390/su15129626	Sustainability (Switzerland)
Byiringiro, S.; Lacanienta, C.; Clark, R.; Evans, C.; Stevens, S.; Reese, M.; Ouyang, P.; Terkowitz, M.; Weston, C.; Galiatsatos, P.; Guerrero Vazquez, M.; Luthardt, F. W.; Dennison Himmelfarb, C. R.	Digital and virtual strategies to advance community stakeholder engagement in research during COVID-19 pandemic	2022	10.1017/cts.2022.457	Journal of Clinical and Translational Science
Cartwright, C.; Rahman, A.; Islam, S.; Lockyer, B.; Roper, E.; Worcester, M.; Zarate, M.; McEachan, R.; Amini, N.; Hammard, R.; Horner, P.; Iqbal, H.	People powered research: what do communities identify as important for happy and healthy children and young people? A multidisciplinary community research priority setting exercise in the City of Bradford, United Kingdom (UK)	2023	10.1186/s12939-023-01881-y	International Journal for Equity in Health
Chammas, G.; Kayed, S.; Al Shami, A.; Kays, W.; Citton, M.; Kalot, M.; Al Marj, E.; Fakhr, M.; Yehya, N. A.; Talhouk, S. N.; Al-Hindi, M.; Zein-El-Dine, S.; Tamim, H.; Lakkis, I.; Abou Najm, M.; Saliba, N. A.	Transdisciplinary interventions for environmental sustainability	2020	10.1016/j.wasman.2020.03.043	Waste Management
Chantziaras, I.; Van Meensel, J.; Hoschet, I.; Leen, F.; Messely, L.; Maes, D.; Millet, S.	Carcass gain per kg feed intake: developing a stakeholder-driven benchmark for comparing grow-finishing pig performance	2020	10.1017/s1751731120001664	Animal
Chesla, Catherine A.; Chun, Kevin M.; Kwan, Christine M. L.; Mullan, Joseph T.; Kwong, Yulanda; Hsu, Lydia; Huang, Peggy; Strycker, Lisa A.; Shum, Tina; To, Diana; Kao, Rudy; Waters, Catherine M.	Testing the efficacy of culturally adapted coping skills training for Chinese American immigrants with type 2 diabetes using community-based participatory research	2013	10.1002/nur.21543	Research in Nursing & Health
Cheung, S. Y. S.; Lei, D.; Chan, F. Y. F.; Tieben, H.	Public Space Usage and Wellâ€ Being: Participatory Action Research With Vulnerable Groups in Hyperâ€ Dense Environments	2022	10.17645/up.v7i4.5764	Urban Planning
Coleman, K.; Allen, C.; Eslan, A.; Shepherd, C.; Sanchez, J.	Building Team-based Primary Care: Lessons From an Academicâ€“Community Network Partnership	2023	10.1353/cpr.2023.a914124	Progress in Community Health Partnerships: Research, Education, and Action
Croisant, S. A.; Lin, Y. L.; Shearer, J. J.; Prochaska, J.; Phillips-Savoy, A.; Gee, J.; Jackson, D.; Panettieri, R. A., Jr.; Howarth, M.; Sullivan, J.; Black, B. J.; Tate, J.; Nguyen, D.; Anthony, A.; Khan, A.; Fernando, H.; Shakeel Ansari, G. A.; Rowe, G.; Howrey, B.; Singleton, C.; Elferink, C.	The gulf coast health Alliance: health risks related to the macondo spill (GC-HARMS) study: Self-reported health effects	2017	10.3390/ijerph14111328	International Journal of Environmental Research and Public Health
Dada, S.; McKay, G.; Mateus, A.; Lees, S.	Lessons learned from engaging communities for Ebola vaccine trials in Sierra Leone: reciprocity, relatability, relationships and respect (the four R’s)	2019	10.1186/s12889-019-7978-4	BMC Public Health
Dawes, Glenn; Davidson, Andrea; Walden, Edward; Isaacs, Sarah	Keeping on Country: Understanding and Responding to Crime and Recidivism in Remote Indigenous Communities	2017	10.1111/ap.12296	Australian Psychologist
De Brún, T., M. O’Reilly-de Brún, E. Van Weel-Baumgarten, N. Burns, C. Dowrick, C. Lionis, C. O’donnell et al.	Using participatory learning & action (PLA) research techniques for inter-stakeholder dialogue in primary healthcare: An analysis of stakeholders™ experiences	2017	10.1186/s40900-017-0077-8	Research Involvement and Engagement
Dontje, M. L.; Kruitwagen-van Reenen, E.; van Wijk, E.; Baars, E.; Visser-Meily, J. M. A.; Beelen, A.; van Os, J.; van den Berg, L.; van der Meijden, C.; Cornelissen, V.; Eimers, M.; Horemans, A.; Kruitwagen, E.; Sterk, J.; Peeters, L.; Pirard, E.; Spendel, T.; Koopman, A. W.; Timmermans, R.; Kramer, G.; Schouten, E.; Focks, R. J.	Evaluation of the nation-wide implementation of ALS home monitoring & coaching: an e-health innovation for personalized care for patients with motor neuron disease	2022	10.1186/s12913-022-08724-6	BMC Health Services Research
Flint, C. G.; Robinson, E. S.; Kellogg, J.; Ferguson, G.; BouFajreldin, L.; Dolan, M.; Raskin, I.; Lila, M. A.	Promoting wellness in Alaskan villages: Integrating traditional knowledge and science of wild berries	2011	10.1007/s10393-011-0707-9	Ecohealth
Hiratsuka, V. Y.; Trinidad, S. B.; Ludman, E. J.; Shaw, J. L.; Burke, W.; Robinson, R. F.; Dillard, D. A.	“You Actually View Us as the Experts in Our Own System:” Indigenous-Academic Community Partnership	2020	10.1353/cpr.2020.0018	Prog Community Health Partnersh
Hove, J.; Mabetha, D.; van der Merwe, M.; Twine, R.; Kahn, K.; Witter, S.; D’Ambruoso, L.	Participatory action research to address lack of safe water, a community-nominated health priority in rural South Africa	2023	10.1371/journal.pone.0288524	PLoS ONE
Ingram, M.; Marrone, N.; Sanchez, D. T.; Sander, A.; Navarro, C.; de Zapien, J. G.; Colina, S.; Harris, F.	Addressing Hearing Health Care Disparities among Older Adults in a US-Mexico Border Community	2016	10.3389/fpubh.2016.00169	Front Public Health
Jeanjean, M.; Lees, J.; Allen, B. L.; Cohen, A. K.	Interdisciplinary community-based participatory health research across the industrial region of the Ã‰tang de Berre: The EPSEAL Fos Crau study	2021	10.1016/j.respe.2021.04.141	Revue d’Epidemiologie et de Sante Publique
Kombo, B. K.; Thomann, M.; Musyoki, H.; Olango, K.; Kuria, S.; Kyana, M.; Otieno, M.; Njiraini, M.; Musimbi, J.; Bhattacharjeea, P.; Lorway, R.; Lazarus, L.	From collaborator to colleague: a community-based program science approach for engaging Kenyan communities of gay, bisexual and other men who have sex with men in HIV research	2023	10.1080/09581596.2023.2260935	Critical Public Health
Larson, K. L., Hansen, C., Ritz, M., & Carreño, D.	Acceptance and Impact of Point-of-Use Water Filtration Systems in Rural Guatemala	2017	10.1111/jnu.12260	J Nurs Scholarsh
Larson, K. L.; Mathews, H. F.; Melendez, C. R.; Hupp, T.; Estrada, M.; Moye, J. P.; Passwater, C. C.; Muzaffar, M.	Original Research: Can a Palliative Care Lay Health Advisor-Nurse Partnership Improve Health Equity for Latinos with Cancer?	2023	10.1097/01.Naj.0000944912.42194.33	Am J Nurs
Lebow-Skelley, E.; Young, L.; Noibi, Y.; Blaginin, K.; Hooker, M.; Williamson, D.; Tomlinson, M. S.; Kegler, M. C.; Pearson, M. A.	Defining the Exposome Using Popular Education and Concept Mapping With Communities in Atlanta, Georgia	2022	10.3389/fpubh.2022.842539	Front Public Health
Macarow, K.; Hilton, R.; Coombs, G.	Hands across Care: Art and social practice in health and elder care contexts	2021	10.1016/j.puhe.2021.03.024	Public Health
Maciver, D.; Prior, S.; Forsyth, K.; Walsh, M.; Meiklejohn, A.; Irvine, L.; Pentland, D.	Vocational rehabilitation: Facilitating evidence based practice through participatory action research	2013	10.3109/09638237.2012.734659	Journal of Mental Health
MacLeod, M. L. P.; Leese, J.; Garraway, L.; Oelke, N. D.; Munro, S.; Bailey, S.; Hoens, A. M.; Loo, S.; Valdovinos, A.; Wick, U.; Zimmer, P.; Li, L. C.	Engaging with patients in research on knowledge translation/implementation science methods: a self study	2022	10.1186/s40900-022-00375-5	Res Involv Engagem
Madison, S.; Colon-Moya, A. D.; Morales-Cosme, W.; Lorenzi, M.; Diaz, A.; Hickson, B.; Monteiro, K.; Muniz Ruiz, A.; Perez, A.; Redondo, R.; Reid, D.; Robles, J.; Santiago, M.; Thompson, O.; Wade, J.; White, M.; Castillo, G.; Valenzuela, C.	Evolution of a research team: the patient partner perspective	2022	10.1186/s40900-022-00377-3	Research Involvement and Engagement
Madouas, M.; Henaux, M.; Delrieu, V.; Jaugey, C.; Teillet, E.; Perrin, M.; Schmitt, C.; Oberheiden, M.; Schermesser, F.; Soustre-Gacougnolle, I.; Masson, J. E.	Learning, reflexivity, decision-making, and behavioral change for sustainable viticulture associated with participatory action research	2023	10.1057/s41599-023-01690-2	Humanities and Social Sciences Communications
Martin, L.; Gupta, M.; Maass, K. L.; Melander, C.; Singerhouse, E.; Barrick, K.; Samad, T.; Sharkey, T. C.; Ayler, T.; Forliti, T.; Friedman, J.; Nelson, C.; Sortillion, D.	Learning Each Otherâ€™s Language and Building Trust: Community-Engaged Transdisciplinary Team Building for Research on Human Trafficking Operations and Disruption	2022	10.1177/16094069221101966	International Journal of Qualitative Methods
Masson, J. E.; Soustre-Gacougnolle, I.; Perrin, M.; Schmitt, C.; Henaux, M.; Jaugey, C.; Teillet, E.; Lollier, M.; Lallemand, J. F.; Schermesser, F.; Isner, P.; Schaeffer, P.; Koehler, C.; Rominger, C.; Boesch, M.; RuÃ©, P.; Miclo, Y.; Bursin, A.; Dauer, E.; Hetsch, J. M.; Burgenath, M.; Bauer, J.; Breuzard, M.; MurÃ©, V.; Cousin, F.; LassabliÃ¨re, R.; Giee Westhalten	Transdisciplinary participatory action research from questions to actionable knowledge for sustainable viticulture development	2021	10.1057/s41599-020-00693-7	Humanities and Social Sciences Communications
Mastrocinque, J. M.; Cerulli, C.; Thew, D.; Chin, N. P.; Pollard, R. Q.	Understanding Intimate Partner Violence Perpetration Involving the Deaf Population	2022	10.1177/0886260520916265	J Interpers Violence
Masunaga, Y.; Jaiteh, F.; Manneh, E.; Balen, J.; Okebe, J.; D’Alessandro, U.; Nieto-Sanchez, C.; de Vries, D. H.; Gerrets, R.; Peeters Grietens, K.; Muela Ribera, J.	The Community Lab of Ideas for Health: Community-Based Transdisciplinary Solutions in a Malaria Elimination Trial in The Gambia	2021	10.3389/fpubh.2021.637714	Front Public Health
Matthew, R. A.; Orpinas, P.; Calva, A.; Bermudez, J. M.; Darbisi, C.	Lazos Hispanos: Promising Strategies and Lessons Learned in the Development of a Multisystem, Community-Based Promotoras Program	2020	10.1007/s10935-020-00587-z	J Prim Prev
Moore, J.	Postmigration Living Difficulties, Help-Seeking and Community Resilience in the Initial Stages of Migration: Coproducing Community Practice with Recent Irish Migrants to London	2018	10.1080/10705422.2018.1450319	Journal of Community Practice
Motta-Ochoa, R.; Bresba, P.; Da Silva Castanheira, J.; Lai Kwan, C.; Shaffer, S.; Julien, O.; William, M.; Blain-Moraes, S.	“When I hear my language, I travel back in time and I feel at home:” Intersections of culture with social inclusion and exclusion of persons with dementia and their caregivers	2021	10.1177/13634615211001707	Transcult Psychiatry
Nava, M.; English, A. S.; Fulmer, L.; Sanchez, K.	An action research partnership in an urban Texas county to explore barriers and opportunities for collaborative community health needs assessments	2023	10.3389/fpubh.2023.1244143	Frontiers in Public Health
Nix, E.; Paulose, J.; Shrubsole, C.; Altamirano-Medina, H.; Belesova, K.; Davies, M.; Khosla, R.; Wilkinson, P.	Participatory Action Research as a Framework for Transdisciplinary Collaboration: A Pilot Study on Healthy, Sustainable, Low-Income Housing in Delhi, India	2019	10.1002/gch2.201800054	Glob Chall
Nwakoby, C.; Pierce, L. J.; Crawford, R.; Conserve, D.; Perkins, J.; Hurt, S.; Ahonkhai, A. A.	Establishing an Academicâ€“Community Partnership to Explore the Potential of Barbers and Barbershops in the Southern United States to Address Racial Disparities in HIV Care Outcomes for Black Men Living With HIV	2023	10.1177/15579883231152114	American Journal of Men’s Health
O’Reilly-de Brún, M., de Brún, T., Okonkwo, E., Bonsenge-Bokanga, J. S., De Almeida Silva, M. M., Ogbebor, F., Mierzejewska, A., Nnadi, L., van Weel-Baumgarten, E., van Weel, C., van den Muijsenbergh, M., & MacFarlane, A.	Using Participatory Learning & Action research to access and engage with ‘hard to reach’ migrants in primary healthcare research	2016	10.1186/s12913-015-1247-8	BMC Health Services Research
Pinsoneault, L. T.; Connors, E. R.; Jacobs, E. A.; Broeckling, J.	Go Slow to Go Fast: Successful Engagement Strategies for Patient-Centered, Multi-Site Research, Involving Academic and Community-Based Organizations	2019	10.1007/s11606-018-4701-6	J Gen Intern Med
Prochaska, J. M.; Mauriello, L.; Dyment, S.; Gökbayrak, S.	Designing a health behavior change program for dissemination to underserved pregnant women	2011	10.1111/j.1525-1446.2011.00959.x	Public Health Nursing
Reed, H.; Langley, J.; Stanton, A.; Heron, N.; Clarke, Z.; Judge, S.; McCarthy, A.; Squire, G.; Quinn, A.; Wells, O.; Tindale, W.; Baxter, S.; Shaw, P. J.; McDermott, C. J.	Head-Up; An interdisciplinary, participatory and co-design process informing the development of a novel head and neck support for people living with progressive neck muscle weakness	2015	10.3109/03091902.2015.1088092	Journal of Medical Engineering and Technology
Ridde, V.; Yaogo, M.; Kafando, Y.; Kadio, K.; Ouedraogo, M.; Sanfo, M.; Coulibaly, N.; Bicaba, A.; Haddad, S.	Challenges of scaling up and of knowledge transfer in an action research project in Burkina Faso to exempt the worst-off from health care user fees	2011	10.1186/1472-698X-11-S2-S9	BMC International Health and Human Rights
Rivera-Díaz, M., Correa-Luna, J., Álamo-Rodríguez, N. M., Barreto-Cortés, E., Paz-Zayas, V., Martínez-Avilés, M. D. L., Muñoz-Sosa, N., López-Ortiz, M.T., Ortiz-Ortiz, Y., Pizarro-Claudio, D., Reyes-Gil, Y & Tejada-Duarte, R.	Somos Dign@s and Trayecto Dignidad: a National Campaign for Advocating Human Rights in Puerto Rico	2021	10.1007/s41134-021-00175-z	Journal of Human Rights and Social Work
Salma, J.; Giri, D.	Engaging Immigrant and Racialized Communities in Community-Based Participatory Research During the COVID-19 Pandemic: Challenges and Opportunities	2021	10.1177/16094069211036293	International Journal of Qualitative Methods
Sangalang, C. C.; Chen, A. C.; Kulis, S. S.; Yabiku, S. T.	Development and Validation of a Racial Discrimination Measure for Cambodian American Adolescents	2015	10.1037/a0036706	Asian Am J Psychol
Schodl, K.; Leeb, C.; Winckler, C.	Developing scienceâ€“industry collaborations into a transdisciplinary process: a case study on improving sustainability of pork production	2015	10.1007/s11625-015-0329-1	Sustainability Science
Scruby, L. S.; Canales, M. K.; Ferguson, E.; Gregory, D.	Promoting Face-to-Face Dialog for Community Engagement in a Digital Age	2017	10.1177/0844562117726939	The Canadian journal of nursing research = Revue canadienne de recherche en sciences infirmieres
Sprague Martinez, L.; Reisner, E.; Campbell, M.; Brugge, D.	Participatory Democracy, Community Organizing and the Community Assessment of Freeway Exposure and Health (CAFEH) Partnership	2017	10.3390/ijerph14020149	Int J Environ Res Public Health
Street, J.; Baum, F.; Anderson, I.	Developing a collaborative research system for Aboriginal health	2007	10.1111/j.1753-6405.2007.00090.x	Australian and New Zealand Journal of Public Health
Sutherland, C.; Reynaert, E.; Sindall, R. C.; Riechmann, M. E.; Magwaza, F.; Lienert, J.; Buthelezi, S.; Khumalo, D.; Dhlamini, S.; Morgenroth, E.; Udert, K. M.	Innovation for improved hand hygiene: Field testing the Autarky handwashing station in collaboration with informal settlement residents in Durban, South Africa	2021	10.1016/j.scitotenv.2021.149024	Sci Total Environ
Temple, L.; Kwa, M.; Fogain, R.; Pefoura, A. M.	Participatory determinants of innovation and their impact on plantain production systems in Cameroon	2006	10.1080/14735903.2006.9684804	International Journal of Agricultural Sustainability
Veisi, H.; Jackson-Smith, D.; Arrueta, L.	Alignment of stakeholder and scientist understandings and expectations in a participatory modeling project	2022	10.1016/j.envsci.2022.04.004	Environmental Science and Policy
Vingilis, E.; Hartford, K.; Schrecker, T.; Mitchell, B.; Lent, B.; Bishop, J.	Integrating knowledge generation with knowledge diffusion and utilization: a case study analysis of the Consortium for Applied Research and Evaluation in Mental Health	2003	10.1007/bf03405087	Can J Public Health
Volkov, B. B.; Pulley, C.; Shlafer, R.	Addressing health disparities in the criminal legal system: Translational benefits, challenges, and facilitators of impactful research with incarcerated pregnant women	2023	10.1017/cts.2023.528	Journal of Clinical and Translational Science
Ward, V.; Pinkney, L.; Fry, G.	Developing a framework for gathering and using service user experiences to improve integrated health and social care: The SUFFICE framework	2016	10.1186/s13104-016-2230-0	BMC Research Notes
Whitehead, D.; Keast, J.; Montgomery, V.; Hayman, S.	A multidisciplinary osteoporosis service-based action research study	2004		Health Education Journal
Wiedemann, R.; Stamm, C.; Staudacher, P.	Participatory knowledge integration to promote safe pesticide use in Uganda	2022	10.1016/j.envsci.2021.11.012	Environmental Science and Policy
Wood, B.; Burchell, A. N.; Escott, N.; Little, J.; Maar, M.; Ogilvie, G.; Severini, A.; Bishop, L.; Morrisseau, K.; Zehbe, I.	Using community engagement to inform and implement a community-randomized controlled trial in the Anishinaabek cervical cancer screening study	2014	10.3389/fonc.2014.00027	Frontiers in Oncology
Wu, E.; Villani, J.; Davis, A.; Fareed, N.; Harris, D. R.; Huerta, T. R.; LaRochelle, M. R.; Miller, C. C.; Oga, E. A.	Community dashboards to support data-informed decision-making in the HEALing communities study	2020	10.1016/j.drugalcdep.2020.108331	Drug Alcohol Depend
Yashadhana, A.; Fields, T.; Burnett, A.; Zwi, A. B.	Reexamining the gap: A critical realist analysis of eye health inequity among Aboriginal and Torres Strait Islander Australians	2021	10.1016/j.socscimed.2021.114230	Social Science and Medicine

### Funding

Fifty-seven (85.1%) studies were supported by external funding only and eleven (16.4%) were supported by internal institutional funds. Twelve (17.9%) studies were supported by multiple funding sources (Appendix 3). Eleven (16.4%) studies did not list funding sources; none explicitly reported not having been funded.

### Topics, study populations, and settings

The studies included in this review included researchers and community partners in diverse settings. Most studies focused on partnerships created to address human health (e.g., chronic and infectious disease, cancer) and its determinants (e.g., hygiene, housing, poverty, climate change, nutrition security). All but three studies (95.5%) focused on human populations with shared experiences (e.g., certain health conditions, members of the same community) or groups of people, such as research teams. Six (9.0%) studies focused on non-human populations or topics, (e.g., animals, agriculture, environmental health; four of these focused on both human and non-human populations/topics). Studies took place in specific geographic or cultural communities (74.6%), health centers (16.4%), research centers and institutes (16.4%), schools (3.0%), or a combination of these settings (10.4%). Thirty-four (50.7%) studies were conducted in North America; of those, 28 were conducted in the U.S., 7 in Canada, 3 in Guatemala and 2 in Mexico. Twenty-four (35.8%) were conducted in Europe, 7 (10.4%) in Africa, 3 (4.5%) in Asia, and 1 (1.5%) in Australia. Five manuscripts (7.5%) described multi-country studies.

### Theoretical frameworks and approaches

Over half (65.7%) of studies described having used one or more theoretical frameworks or approaches. Studies reported using behavior change theories (e.g., Theory of Planned Behavior, Social Cognitive Theory, Health Belief Model); participatory action frameworks; Community-based participatory research (CBPR); ecological theory; design theory; and transdisciplinary research frameworks.

### Methods and outcomes

Nearly two-thirds (60.9%) of studies utilized qualitative methods (e.g., interviews, focus groups, observations); 26.1% utilized quantitative methods (e.g., descriptive, non-randomized, randomized controlled trial); 47.8% used mixed or multiple methods. The majority (80.6%) of studies assessed at least one outcome and 15 (22.4%) assessed partnership outcomes specifically. Partnership outcomes included quantitative measures and qualitative narratives regarding topics such as challenges to partnership, new social ties (e.g., collaborations between groups or individuals who had not previously interacted), characteristics of collaborative culture, power relations, trust and trust building, bidirectional learning, equity in research participation, and research team well-being.

### Community engagement

The studies included in this review were examined for their explicit description of 16 community engagement characteristics (Table 3) [2,4,37–39] The greatest number of studies (*n* = 56; 83.6%) described an asset-based approach wherein all collaborators’ strengths and knowledge were respected and incorporated. Studies described community member roles on the study team as participating in implementation (*n* = 47 studies, 70.1%); data analysis (*n* = 47, 70.1%); co-defining the issue (*n* = 43, 64.2%); and co-designing the study (*n* = 38, 56.7%). Over half (61.2%) explicitly described a process of reporting findings back to the community through activities such as town halls, community meetings, and reports written in plain language. Conversely, less than a third (*n* = 22, 32.8%) described having included community members as coauthors on publications, presentations, or reports of findings. Less than half of studies (41.8%) explicitly described their work as focusing on a community identified priority; 26 (38.8%) described ongoing commitment to community members beyond a single funded project, and 14 (20.9%) described paying community members as part of their study teams. The specific type of community partners engaged varied across research studies. In 37 (55.2%) studies, authors described having engaged individuals impacted by the topic addressed (e.g., disease, perceptions of health, partnership, environmental health); 35 (52.2%) described partnering with members of community organizations and 25 (37.3%) described partnering with leaders of community organizations. More than half (52.2%) described collaborating across multiple community partner types.

**Table 3.** Community engagement and team science characteristics described in empirical studies utilizing both community engagement and team science

Community engagement characteristic	n	%
Asset-based approach	56	83.6%
Policy implications	54	80.6%
Reciprocity	53	79.1%
Study implementation	47	70.1%
Data analysis	47	70.1%
Co-defining the issue	43	64.2%
Communicating study findings	41	61.2%
Study co-design	38	56.7%
Instrument co-design	29	43.3%
Sustainability	29	43.3%
Addressing community priority	28	41.8%
Advisory boards	27	40.3%
Ongoing commitment to community	26	38.8%
Building community capacity	24	35.8%
Coauthors	22	32.8%
Community member compensation	14	20.9%
Team science characteristic	n	%
Team partnership goals	56	83.6%
Leveraging expertise	55	82.1%
Organizational context	52	77.6%
Team member roles	51	76.1%
Team research management	46	68.7%
Team processes	41	61.2%
Communication	36	53.7%
Collaborative problem-solving	33	49.3%
Material resources	31	46.3%
Team leadership	24	35.8%
Facilitating team affect	21	31.3%
Interpersonal team effectiveness	18	26.9%
Manuscript focus	n	%
Community engagement	39	58.2%
Team science	6	9.0%
Equal emphasis on both	22	32.8%
Community representatives	n	%
Individuals impacted	37	55.2%
Members of specific organizations	35	52.2%
Leaders of specific organizations	25	37.3%
Community leaders	17	25.4%
Other	12	17.9%
More than one of these representatives	35	52.2%

### Team science

Studies in this review were assessed for their description of 12 team science characteristics (Table 3) [40,42,43]. The greatest number of studies (*n* = 56, 83.6%) described team partnership goals, such as engaging in bidirectional learning; building capacity of researchers and community partners; conducting outreach, educational, and reflexive efforts among collaborators, and/or identifying and addressing a community priority. Similarly, 51 (76.1%) clearly named team member roles (e.g., identifying the priority issue; co-designing the study and/or data collection instruments; advising) and the same number described how specific roles were needed to leverage the varying expertise of collaborators and 55 (82.1%) described how those roles were necessary to leverage the expertise of different team members. Over three quarters described the organizational context of the team research (*n* = 52, 77.6%), and 46 (68.7%) described team research management. Fewer studies, however, described team leadership (*n* = *n* = 24; 35.8%), the process of facilitating team affect (*n* = 21; 31.3%), or interpersonal team effectiveness (*n* = 18, 26.9%).

### Team science and community engagement

Associations between specific characteristics of community engagement and team science are reported in Table 4. *Reciprocity, co-Defining the issue, sustainability, and ongoing commitment to the community* were the most common community engagement characteristics that were significantly associated with any team science characteristic. *Team research management, material resources,* and *team processes* were the most common team science characteristics that were significantly associated with any community engagement characteristics. Describing *reciprocity*, a community-engagement characteristic, was significantly associated with describing multiple team science characteristics that include *organizational context, team member roles*, *team processes, team research management, material resources, and collaborative problem-solving*. Describing the community engagement characteristic of *asset-based approach* was significantly associated with describing team science characteristics *team member roles, team processes, team research management, and material resources.* Additionally, describing the community engagement characteristic of *co-defining the issue* was significantly associated with describing the team science characteristics of *team leadership, team partnership goals, team processes, communication, team research management, material resources,* and *collaborative problem-solving*. No significant associations were found between any team science characteristic and the community engagement characteristics of *instrument co-design, study implementation, policy implications,* or *advisory boards.* No significant associations were found between any community engagement characteristic and *interpersonal team effectiveness*.

**Table 4.** Significant associations between community engagement and team science characteristics described in empirical studies utilizing both community engagement and team science

Community engagement characteristic		Team science characteristic	
1	Reciprocity	1	Organizational context**
		4	Team member roles*
		6	Team processes*
		10	Team research management*
		11	Material resources**
		12	Collaborative problem-solving*
2	Asset-based approach	4	Team member roles**
		6	Team processes**
		10	Team research management***
		11	Material resources*
3	Co-defining the issue	2	Team leadership**
		3	Team partnership goals**
		6	Team processes***
		9	Communication***
		10	Team research management***
		11	Material resources**
		12	Collaborative problem-solving**
4	Addressing community priority	12	Collaborative problem-solving*
5	Study co-design	4	Team member roles**
8	Data analysis	4	Team member roles**
		5	Leveraging expertise*
9	Coauthors	1	Organizational context**
		9	Communication*
		11	Material resources*
10	Communicating study findings	1	Organizational context*
		6	Team processes*
		9	Communication*
		10	Team research management**
		11	Material resources*
12	Sustainability	1	Organizational context*
		2	Team leadership*
		3	Team partnership goals*
		5	Leveraging expertise*
		6	Team processes**
		10	Team research management*
13	Community member compensation	9	Communication*
		10	Team research management*
		12	Collaborative problem-solving*
15	Ongoing commitment to community	1	Organizational context*
		2	Team leadership*
		3	Team partnership goals*
		6	Team processes*
		8	Facilitating team affect*
		11	Material resources*
16	Building community capacity	3	Team partnership goals*
		11	Material resources*

No significant associations between any team science characteristic and these community engagement characteristics: instrument co-design, study implementation, policy implications, advisory boards. No significant association between any community engagement characteristic and interpersonal team effectiveness.

*p < 0.05, ** *p* < 0.01, ***p < 0.001.

## Discussion

This manuscript is the first, to our knowledge, to use a scoping review approach to interrogate the intersection of the community engagement and team science literature and provides a foundation for further research in this area. The articles included in this scoping review indicate that publication of empirical studies utilizing both community engagement and team science has been increasing since 2004. This finding is consistent with reviews that found that research studies in community engagement and CBPR have proliferated [2,17,18]. This growth in interest in team science and community engagement could be a result of an evolving awareness of team functioning and approaches to both partnership *and* community engaged research in response to COVID-19, or perhaps a widescale desire to reimagine community-engaged research as an approach to social change [27,48,49]. Most studies in our review focused on partnering with both community members and community organizations to ameliorate the health of systematically marginalized communities; however, several studies also utilized team science and community engagement strategies to address topics such as environmental health, agriculture, and fisheries, topics not commonly addressed in community-engagement research. Just under half (42%) of studies were conducted outside of the U.S.

More than three-quarters of studies described community-engagement characteristics such as taking an *asset-based approach, focusing on policy implications of the research that will benefit the community,* and *collaboration grounded in bidirectional exchange.* Over half outlined specific roles of communities as part of the research team, such as *co-defining the issue* and *co-designing the study* or *analyzing data*. Unsurprisingly, the team science characteristics most often described in these studies, such as *team partnership goals, leveraging expertise of all team members, team member roles,* and *organizational context* align with principles of community engagement. However, the fact that no significant associations were found between any team science characteristic and the community engagement characteristics *instrument co-design, study implementation, policy implications,* or *advisory boards* suggests that these factors may be characteristics unique to community engagement, or that they represent a gap in the team science literature.

With the exceptions described above, this review revealed inconsistent and incomplete reporting of community engagement and team science characteristics. Informed by the synergies as well as the inconsistencies and gaps in the literature, we developed recommendations for research teams, research institutions, journals, and funding bodies that we expect will enhance existing efforts and better support and strengthen this important and emerging approach to research (Table 5). For example, less than 25% of studies reported compensating community members of the study team, which may reflect institutional practices. Equitable collaboration across community and academic partners requires equitable compensation [50]; yet, few academic institutions have policies in place to compensate community partners. Accordingly, we recommend that institutions create formal policies, guidelines, and processes that facilitate timely compensation for community members who contribute to research teams.

**Table 5.** Recommendations for research teams, research institutions, journals, and funding bodies

Finding	Recommendations	Target audience
Fewer than 25% of studies described this community engagement characteristic: Community member compensation*	Create formal policies, guidelines and processes that facilitate timely compensation for community members who contribute to research teams	Research institutions
	Pay community members to participate in advisory boards and/or as members of/staff on the research team	Research teams
Fewer than half of studies described these community engagement and team science characteristics: Instrument co-design* Sustainability* Addressing community priority* Advisory boards* Ongoing commitment to community* Building community capacity Coauthors* Collaborative problem-solving** Material resources** Team leadership** Facilitating team affect** Interpersonal team effectiveness**	Release funding notice with sufficient time to allow for meaningful community engagement and integration of community’s input in the planning of the proposal	Funding bodies
	Fund trials that investigate different types of community engagement and team science strategies to continue to build the science of community engagement and team science	Funding bodies
	Provide funding to foster formation of community engaged collaborations, including mechanisms to sustain collaborations over time, incentivize community identified questions, develop team leadership, and enhance team communication	Funding bodies / Research institutions
	Refine grant requirements so applicants can describe community engagement and team science approaches (e.g., basic definitions, training, criteria)	Funding bodies
	Include explicit, scored sections of grant applications that address integrated community engagement and team science approaches (e.g., teaming plans, community engagement plans)	Funding bodies
	Consider offering longer funding periods or the ability to renew if community engaged work is going well based on metrics defined in the grant requirements	Funding bodies
	Pay community members to participate as study section/grant reviewers	Funding bodies
	Integrate community perspectives in producing the manuscript and acknowledge various individuals who may not have formally satisfied requirements to be named as authors	Research teams
	Require a technical paper describing team and community engagement processes, including aspects such as team formation, decision-making, communication, and addressing conflict	Journals
	Create guidelines and a checklist (similar to COREQ or GRIPP) for reporting community engagement and team science approaches. For example, authors may indicate whether and how the study: Is multi-, inter-, or transdisciplinary Utilizes team science, and in what ways (e.g., describe teaming and communication plan, describe plan for reporting and disseminating results) ￼ Engages the community, and at what level (e.g., community members, organizations, etc.) and through what means Co-produces and disseminates results of research	Journals
Between 50-74% of studies described these community engagement and team science characteristics: Study implementation* Data analysis* Co-defining the issue* Communicating study findings* Study co-design* Team research management** Team processes** Communication**	Articulate and assess processes (e.g., collaboration planning) that explicate all team member roles, clearly describe team processes, facilitate collaboration and effective communication between partners throughout and beyond the study period	Research teams
	Incorporate education and training on team science and community engaged approaches into curricula	Research institutions
	Recruit reviewers who conduct community engagement and team science research and build capacity of all reviewers to understand what authentic community engagement entails and the team science approaches that can support this engagement	Funding bodies
	Invite publications on methods for conducting community engagement and team science approaches	Journals
More than 75% of studies described these community engagement and team science characteristics: Asset-based approach* Policy implications* Reciprocity/bidirectional exchange* Team partnership goals** Leveraging expertise** Organizational context** Team member roles**	Authentically engage community partners as content experts in identifying the topic to address, study design, implementation, and data interpretation; this can be done through assembling community advisory boards	Research teams
	Equitably engage in power sharing and decision-making during the planning, implementation, and dissemination of the study	Research teams
	Continue to support and expand activities that facilitate reciprocity and bidirectional exchange. This could occur by moving from emulating academic roles and responsibilities to applying community-led frameworks to foster participation from communities. Such an approach could advance both fields of community engagement and team science individually as well as an integrated community engagement-team science approach	Research teams

*Community engagement characteristic.

**Team science characteristic.

Addressing a community identified priority is foundational to CBPR [37], a principle that could be facilitated by convening advisory boards comprised of community members [9,51], and by partnering to develop collaboration and teaming plans [52]. Yet, fewer than half of the studies in this review described having convened an advisory board or focusing on a need or priority identified by the community. Moreover, fewer than half described an ongoing commitment to the community. Few studies described building community capacity that could be transferrable to future projects and other priorities identified by the community or an ongoing commitment to the community beyond any single funded project, characteristics that are emphasized in CBPR [37]. These practices – or lack thereof – could be a result of the imbalance of the priorities of funders of traditional research, as well as the design of research and academic institutions that don’t always facilitate or support collaborative work in general and community-engaged teamwork and collaboration specifically. For example, evidence shows that CTSA hubs consistently struggle to align community priorities with researcher expertise, academic institution priorities, and funding structures [53,54]. A study of CTSA institutions also found that community and industry partners perceived that academic institutions lack relevant protocols for engaging community partners, including little or no funding for compensating community members [55]. In addition to challenges related to aligning institutional and community priorities, funding agencies’ definitions of community engagement vary considerably, and most agencies do not explicitly define team science, factors that may lead to inconsistency in reporting, measuring, and integrating domains of the two fields. To address these gaps, we recommend that funding notices be released with sufficient time to allow for meaningful community engagement and integration of community’s input in the planning of the proposal. Moreover, we suggest that funding bodies and research institutions designate funds to form community engaged collaborations, including mechanisms to sustain collaborations over time, incentivize community identified questions, develop team leadership, and enhance team communication. Including explicit, scored sections of grant applications could encourage applicants to design research projects that intentionally integrate community engagement and team science approaches, including teaming plans and community engagement plans. We recommend that journals call for technical papers to describe team and community engagement processes, including aspects such as team formation, decision-making, communication, and addressing conflict.

Overall, team science characteristics were less often described than community engagement characteristics, particularly *material resources, team leadership,* and *interpersonal team effectiveness*, all characteristics foundational to team science [40]. Collaboration Planning – a participatory team science approach wherein partners organize their collaboration, identify influences on the collaboration, and strategize to work within those influences [52] – may benefit teams as they integrate team science and community engagement approaches, but few studies referenced such a tool. This absence could be because team science is a relatively nascent field compared to community engaged research, and researchers may be applying team science approaches without knowing the language to describe them. These findings highlight the significance of this work for advancing team science research in general, as well as the Science of Team Science [7]. Moreover, community engagement necessitates team collaboration, but team science does not necessitate community involvement. Applying characteristics and practices from both fields simultaneously has the potential to advance each approach individually and collectively. This is especially relevant as some institutions include both team science and community engagement as part of new appointment, promotion, and tenure policies and faculty codes. Some of the teaming methods characteristic of team science, such as creating communication plans and team agreements, may help those who are or plan to conduct more community engaged research. Similarly, formally applying principles of community engagement, such as identifying and addressing priority issues identified by communities, focusing on ongoing commitment to the community, and sharing study findings could enhance existing team science approaches.

Recognizing that operationalization of engagement fluctuates based on communities, situations, and topics, we have attempted to describe the ways in which communities were engaged and team science approaches were implemented in each research study. However, measuring the extent to which these activities occurred was difficult as it was not always well documented. Authors likely chose which elements of their study to describe based on journal requirements, including word counts and reporting criteria. To our knowledge, although the National Research Council created policy recommendations and guidance for effectively conducting team science in 2015 [7], no standardized reporting requirements exist for community engaged or team science research. The inconsistent and incomplete reporting of community engaged and team science characteristics in this body of work suggests that journal guidelines and checklists similar to COREQ [56] or Guidance for Reporting Involvement of Patients and Public [57] would help elucidate and encourage reporting of which, how, and the extent to which researchers incorporate these characteristics in publications describing their work.

*Limitations.* The empirical studies in this review may not include all studies that have utilized team science and community engagement research, as we could only assess the characteristics of each approach based on our conceptualizations of each approach and on what was described. This review did not evaluate journals’ reporting criteria, a factor that is likely to influence how and to what extent community engagement and team science characteristics are reported. Moreover, we developed lists of community engagement and team science characteristics that served as our inclusion criteria, based on existing literature and expertise both within and outside of the team. It’s possible that we missed articles that addressed these topics in ways not included in our criteria. Relatedly, a wide range of terms is used to describe community engagement and team science; most articles described the results and not the processes used to conduct their studies, so in many cases the community engagement and team science approaches were inferred from the text available. As we note in our recommendations, we suggest that journals provide guidance on how to report on community engagement and team science characteristics so they can be better understood (Table 5). We separated characteristics of community engagement and team science for the inclusion and exclusion criteria; however, as we saw in our results, there is an overlap between the characteristics of each approach. We did not assess methodological rigor in the study designs or potential sources of bias, both limitations of the scoping review approach. This work could not assess the influence of research institutions’ faculty codes and appointments, promotion, and tenure guidelines, which likely include a spectrum of language, metrics, and incentives for community engagement and team science. Future research should investigate the influence of such factors on the proliferation of community engagement and team science research. Finally, although we included studies published in a language other than English, we only included those with an English translation available; consequently, our approach may have introduced a language bias.

## Conclusion

Effective community-engaged research depends on equitable partnerships, shared power, and trust among collaborators [14], and effective team science research is reliant on a team’s capacity to achieve its goals [7]. As researchers continue to integrate community engagement and team science, either as a requirement of funding and research institutions or as a means for more equitably and effectively addressing complex, multifactorial societal problems, common criteria and strategies for promoting integration of the two approaches are needed. This review advances our understanding of the intersection of community engagement and team science research, highlighting domains important to this integration. It provides language and characteristics to assist teams in discerning the extent to which their project utilizes approaches of each and highlights the need for relevant documentation criteria. The 19 recommendations for research teams, research institutions, journals, and funding bodies serve to facilitate advancement of the science and practice of the integration of community engagement and team science efforts.

## Supporting information

Hohl et al. supplementary material 1 Hohl et al. supplementary material

Hohl et al. supplementary material 2 Hohl et al. supplementary material

Hohl et al. supplementary material 3 Hohl et al. supplementary material

## Supplementary material

The supplementary material for this article can be found at https://doi.org/10.1017/cts.2024.644.

## References

[1] Caulfield JL , Brenner EF. Resolving complex community problems: applying collective leadership and Kotter’s change model to wicked problems within social system networks. Nonprofit Manag Leadership. 2020;30(3):509–524.

[2] Ortiz K , Nash J , Shea L , et al. Partnerships, processes, and outcomes: a health equity-focused scoping meta-review of community-engaged scholarship. Annu Rev Publ Health. 2020;41(1):177–199. 10.1146/annurev-publhealth-040119-094220 PMC8095013 31922931

[3] Sánchez V , Sanchez-Youngman S , Dickson E , et al. CBPR implementation framework for community-academic partnerships. Am J Commun Psychol. 2021;67(3-4):284–296. 10.1002/ajcp.12506 33823072

[4] Wallerstein N , Oetzel JG , Sanchez-Youngman S , et al. Engage for equity: a long-term study of community-based participatory research and community-engaged research practices and outcomes. Health Educ Behav. 2020;47(3):380–390. 32437293 10.1177/1090198119897075 PMC8093095

[5] Stokols D , Misra S , Moser RP , Hall KL , Taylor BK. The ecology of team science: understanding contextual influences on transdisciplinary collaboration. Am J Prev Med. 2008;35(2):S96–S115. 18619410 10.1016/j.amepre.2008.05.003

[6] Hall KL , Vogel AL , Huang GC , et al. The science of team science: a review of the empirical evidence and research gaps on collaboration in science. Am Psychol. 2018;73(4):532–548. 29792466 10.1037/amp0000319

[7] National Research Council. Enhancing the Effectiveness of Team Science. Washington, D.C.: National Academies Press, 2015 26247083

[8] Vogel AL , Hall KL , Fiore SM , et al. The team science toolkit: enhancing research collaboration through online knowledge sharing. Am J Prev Med. 2013;45(6):787–789. 24237924 10.1016/j.amepre.2013.09.001

[9] Hohl SD , Neuhouser ML , Thompson B. Re-orienting transdisciplinary research and community-based participatory research for health equity. J Clin Transl Sci. 2022;6(1):p.e22. 10.1017/cts.2022.15 PMC8922293 35321219

[10] Hohl SD , Knerr S , Gehlert S , et al. Transdisciplinary research outcomes based on the transdisciplinary research on energetics and cancer II initiative experience. Res Evaluat. 2020;30(1):39–50. 10.1093/reseval/rvaa026 PMC8896575 35250193

[11] Forsythe LP , Ellis LE , Edmundson L , et al. Patient and stakeholder engagement in the PCORI pilot projects: description and lessons learned. J Gen Intern Med. 2016;31(1):13–21. 26160480 10.1007/s11606-015-3450-z PMC4700002

[12] Fleurence RL , Forsythe LP , Lauer M , et al. Engaging patients and stakeholders in research proposal review: the patient-centered outcomes research institute. Ann Intern Med. 2014;161(2):122–130. 25023251 10.7326/M13-2412

[13] Holzer J , Kass N. Community engagement strategies in the original and renewal applications for CTSA grant funding. Clinical and Translational Science. 2014;7(1):38–43. 24528898 10.1111/cts.12125 PMC5414461

[14] Brush BL , Mentz G , Jensen M , et al. Success in long-standing community-based participatory research (CBPR) partnerships: a scoping literature review. Health Educ Behav. 2020;47(4):556–568. 31619072 10.1177/1090198119882989 PMC7160011

[15] Shalowitz MU , Isacco A , Barquin N , et al. Community-based participatory research: a review of the literature with strategies for community engagement. Journal of Developmental & Behavioral Pediatrics. 2009;30(4):350–361. 19672162 10.1097/DBP.0b013e3181b0ef14

[16] Harris Nwanyanwu K , Grossetta Nardini HK , Shaughness G , Nunez-Smith M , Newman-Casey P-A. Systematic review of community-engaged research in ophthalmology. Expert Rev Ophthalmol. 2017;12(3):233–241. 29333193 10.1080/17469899.2017.1311787 PMC5759339

[17] Rodriguez Espinosa P , Verney SP. The underutilization of community-based participatory research in psychology: a systematic review. Am J Commun Psychol. 2021;67(3-4):312–326. 10.1002/ajcp.12469 PMC8106689 33165973

[18] Julian McFarlane S , Occa A , Peng W , Awonuga O , Morgan SE. Community-based participatory research (CBPR) to enhance participation of racial/ethnic minorities in clinical trials: a 10-year systematic review. Health Commun. 2022;37(9):1075–1092. 34420460 10.1080/10410236.2021.1943978

[19] Ragavan MI , Thomas KA , Fulambarker A , Zaricor J , Goodman LA , Bair-Merritt MH. Exploring the needs and lived experiences of racial and ethnic minority domestic violence survivors through community-based participatory research: a systematic review. Trauma, Violence, & Abuse. 2020;21(5):946–963. 10.1177/1524838018813204 30501479

[20] Michael SL , Barnes SP , Wilkins NJ. Scoping review of family and community engagement strategies used in school-based interventions to promote healthy behaviors. J School Health. 2023;93(9):828–841. 37670597 10.1111/josh.13367 PMC11181466

[21] Russell K , Rosenbaum S , Varela S , Stanton R , Barnett F. Fostering community engagement, participation and empowerment for mental health of adults living in rural communities: a systematic review. Rural Remote Health. 2023;23(1):1–13. 10.22605/RRH7438 36966523

[22] Campbell JA , Yan A , Egede LE. Community-based participatory research interventions to improve diabetes outcomes: a systematic review. Diabet Educ. 2020;46(6):527–539. 10.1177/0145721720962969 PMC7901040 33353510

[23] Shah SK , Nakagawa M , Lieblong BJ. Examining aspects of successful community-based programs promoting cancer screening uptake to reduce cancer health disparity: a systematic review. Prev Med. 2020;141:106242. 32882299 10.1016/j.ypmed.2020.106242 PMC7704699

[24] Little MM , St Hill CA , Ware KB , et al. Team science as interprofessional collaborative research practice: a systematic review of the science of team science literature. J Invest Med. 2017;65(1):15–22. 10.1136/jim-2016-000216 PMC5284346 27619555

[25] Liu Y , Wu Y , Rousseau S , Rousseau R. Reflections on and a short review of the science of team science. Scientometrics. 2020;125(2):937–950.

[26] Ghamgosar A , Nemati-Anaraki L , Panahi S. Barriers and facilitators of conducting research with team science approach: a systematic review. BMC Med Educ. 2023;23(1):638. 37670349 10.1186/s12909-023-04619-0 PMC10478305

[27] Wallerstein N , Calhoun K , Eder M , Kaplow J , Wilkins CH. Engaging the community: community-based participatory research and team science. In: Hall KL , Vogel AL , Croyle RT , eds. Strategies for Team Science Success: Handbook of Evidence-Based Principles for Cross-Disciplinary Science and Practical Lessons Learned from Health Researchers. Cham, Switzerland: Springer International Publishing, 2019:123–134.

[28] Mendell AM , Knerich V , Ranwala DD , et al. Team science competencies across the career life course for translational science teams. J Clin Transl Sci. 2024;8(1):1–24. 10.1017/cts.2024.494 PMC11626591 39655023

[29] Rushforth AM , Selker HP. Broadly Engaged Team Science at Tufts Clinical and Translational Science Institute. In: Broadly Engaged Team Science in Clinical and Translational Research. Cham, Switzerland: Springer, 2022:1–7.

[30] Selker HP , Wilkins CH. From community engagement, to community-engaged research, to broadly engaged team science. J Clin Transl Sci. 2017;1(1):5–6. 31660208 10.1017/cts.2017.1 PMC6798217

[31] Munn Z , Pollock D , Khalil H , et al. What are scoping reviews? Providing a formal definition of scoping reviews as a type of evidence synthesis. JBI Evidence Synthesis. 2022;20(4):950–952. 35249995 10.11124/JBIES-21-00483

[32] Levac D , Colquhoun H , O’Brien KK. Scoping studies: advancing the methodology. Implement Sci. 2010;5(1):69. 20854677 10.1186/1748-5908-5-69 PMC2954944

[33] Arksey H , O’Malley L. Scoping studies: towards a methodological framework. Int J Soc Res Method. 2005;8(1):19–32.

[34] Clinical Translational Science Awards Consortium. Clinical translational science awards consortium. Community engagement key function committee task force on the principles of community engagement. Principles commun Engag. 2011;8:43–54.

[35] Evans E , Funes M , Hong H , et al. Defining and measuring community engagement and community-engaged research: clinical and translational science institutional practices. Prog Commun Health Partnersh Res Educ Action. 2018;12(2):145–156. 10.1353/cpr.2018.0034 PMC6237095 30270224

[36] Nooraie RY , Kwan BM , Cohn E , et al. Advancing health equity through CTSA programs: opportunities for interaction between health equity, dissemination and implementation, and translational science. J Clin Transl Sci. 2020;4(3):168–175. 32695484 10.1017/cts.2020.10 PMC7348010

[37] Israel BA , Schulz A , Parker EA , et al. Critical Issues in Developing and Following CBPR Principles. San Francisco, CA: Jossey-Bass, 2008.

[38] Wallerstein N , Duran B. Community-based participatory research contributions to intervention research: the intersection of science and practice to improve health equity. Am J Public Health. 2010;100(S1):S40–S46. 20147663 10.2105/AJPH.2009.184036 PMC2837458

[39] Wallerstein NB , Yen IH , Syme SL. Integration of social epidemiology and community-engaged interventions to improve health equity. Am J Public Health. 2011;101(5):822–830. 21421960 10.2105/AJPH.2008.140988 PMC3076386

[40] Salas E , Shuffler ML , Thayer AL , Bedwell WL , Lazzara EH. Understanding and improving teamwork in organizations: a scientifically based practical guide. Hum Resour Manage. 2015;54(4):599–622.

[41] Shuffler ML , DiazGranados D , Salas E. There’s a science for that: team development interventions in organizations. Curr Dir Psychol Sci. 2011;20(6):365–372.

[42] Lotrecchiano GR , DiazGranados D , Sprecher J , et al. Individual and team competencies in translational teams. J Clin Transl Sci. 2020;5(1):1–20. 10.1017/cts.2020.551 PMC8057415 33948290

[43] Raue S , Tang S-H , Weiland C , Wenzlik C. The GRPI Model–An Approach for Team Development. Berlin, Germany: White Paper Draft, SE Group, 2013.

[44] Tricco AC , Lillie E , Zarin W , et al. PRISMA extension for scoping reviews (PRISMA-ScR): checklist and explanation. Ann Intern Med. 2018;169(7):467–473. 30178033 10.7326/M18-0850

[45] Harris PA , Taylor R , Minor BL , et al. The REDCap consortium: building an international community of software platform partners. J Biomed Inform. 2019;95:103208. 31078660 10.1016/j.jbi.2019.103208 PMC7254481

[46] Mathes T , Klaßen P , Pieper D. Frequency of data extraction errors and methods to increase data extraction quality: a methodological review. Bmc Med Res Methodol. 2017;17(1):1–8. 29179685 10.1186/s12874-017-0431-4 PMC5704562

[47] National Center for Advancing Translational Sciences. The Collaboration and Engagement Enterprise Committee. https://ccos-cc.ctsa.io/groups/enterprise-committees/collaboration-and-engagement. Accessed September 5, 2024.

[48] Gilmore B , Ndejjo R , Tchetchia A , et al. Community engagement for COVID-19 prevention and control: a rapid evidence synthesis. BMJ Global Health. 2020;5(10):e003188. 10.1136/bmjgh-2020-003188 PMC7554411 33051285

[49] Henry Akintobi T , Jacobs T , Sabbs D , et al. Community engagement of African Americans in the era of COVID-19: considerations, challenges, implications, and recommendations for public health. Prev Chronic Dis. 2020;17:E83. 32790605 10.5888/pcd17.200255 PMC7458103

[50] Langness M , Morgan JW , Cedano S , Falkenburger E. Equitable Compensation for Community Engagement Guidebook. Washington, D.C.: Urban Institute, 2023.

[51] Ortega S , McAlvain MS , Briant KJ , Hohl S , Thompson B. Perspectives of community advisory board members in a community-academic partnership. J Health Care Poor U. 2018;29(4):1529–1543. 10.1353/hpu.2018.0110 PMC6333479 30449761

[52] Hall KL , Vogel AL , Crowston K. Comprehensive collaboration plans: practical considerations spanning across individual collaborators to institutional supports. In: Hall KL, Vogel AL, Croyle RT, eds. Strategies for Team Science Success: Handbook of Evidence-Based Principles for Cross-Disciplinary Science and Practical Lessons Learned from Health Researchers. Cham, Switzerland: Springer International Publishing, 2019: 587–611.

[53] Holzer J , Kass N. Understanding the supports of and challenges to community engagement in the CTSAs. Clin Transl Sci. 2015;8(2):116–122. 25196710 10.1111/cts.12205 PMC4362794

[54] Towfighi A , Orechwa AZ , Aragón TJ , et al. Bridging the gap between research, policy, and practice: lessons learned from academic-public partnerships in the CTSA network. J Clin Transl Sci. 2020;4(3):201–208. 32695489 10.1017/cts.2020.23 PMC7348007

[55] Freeman E , Seifer SD , Stupak M , Martinez LS. Community engagement in the CTSA program: stakeholder responses from a national delphi process. Clin Transl Sci. 2014;7(3):191–5. doi: 10.1111/cts.12158. 24841362 PMC5350819

[56] Booth A , Hannes K , Harden A , Noyes J , Harris J , Tong A. COREQ (consolidated criteria for reporting qualitative studies). In: Guidelines for Reporting Health Research: A User’s Manual. Hoboken, New Jersey: John Wiley & Sons Publishing, 2014:214–226.

[57] Staniszewska S , Brett J , Mockford C , Barber R. The GRIPP checklist: strengthening the quality of patient and public involvement reporting in research. Int J Technol Assess Health Care. 2011;27(4):391–399. doi: 10.1017/s0266462311000481. 22004782

