# Supplementary material for: The intersection of community engagement and team science research: A scoping review
Source: J Clin Transl Sci. 2024 Oct 28;8(1):e208. doi: 10.1017/cts.2024.644 (PMC11713437; doi:10.1017/cts.2024.644)
Supplement: Hohl et al. supplementary material 1 — Hohl et al. supplementary material [file S2059866124006447sup001.docx]

## Appendix 1: Search strategy used in each of the electronic databases

**Team Science/Community Engagement Scoping Review**

**Database Search Terms**

**Pubmed**

**searched 2/28/2022**

**208 results**

(interdisciplinary research[mesh] OR “interdisciplinary research”[tiab] OR “interdisciplinary science”[tiab]  OR “inter-disciplinary research”[tiab] OR “inter-disciplinary science”[tiab] OR  transdisciplinary research[mesh] OR “transdisciplinary science” [tiab]  OR “transdisciplinary research”[tiab]  OR “trans-disciplinary research”[tiab] OR “trans-disciplinary team*”[tiab] OR multidisciplinary research[mesh] OR “multidisciplinary science” [tiab]  OR “multidisciplinary research” [tiab]  OR “multi-disciplinary research*”[tiab] OR “multi-disciplinary science”[tiab] OR “cross-disciplinary research*”[tiab] OR “cross-disciplinary science”[tiab] OR  “cross disciplinary research*” OR “cross disciplinary science”[tiab] OR  “transdisciplinary science” [tiab]  OR “transdisciplinary research” [tiab] OR trans-disciplinary science[tiab] OR “trans-disciplinary research”[tiab] OR multisystem science[tiab]  OR multisystem research[tiab]  OR multi-system science[tiab]  OR multi-system research[tiab]  OR “team science” [tiab]  OR “team science”[ad] OR “team sciences” [tiab]  OR scits[tw] OR “science team”[tiab]  OR “science teams”[tiab]  OR “team research” [tiab]  OR “team based research”[tiab]  OR “team-based research” OR “Translational team*”[tiab] OR “translational research team*”[tiab] OR “interprofessional research”[tiab] OR “group research”[tiab] OR transprofessional research[tiab] OR trans-professional research[tiab]) AND  (Community-Based Participatory Research[Mesh] OR “community based participatory research”[tiab] OR “community-based participatory research”[tiab]  OR CBPR[tiab]  OR  “community engag*”[tiab] OR “community-engag*”[tiab] OR “community based engag*”[tiab] OR “community-based engag*”[tiab] OR “community based research*”[tiab] OR “community based action research*”[tiab] OR “community-based action research”[tiab] OR consumer driven research*[tiab] OR consumer-driven research*[tiab] OR “collaborative public health research*” [tiab] OR “citizen research*”[tiab] OR “participatory research*”[tiab] OR “participatory action research” [tiab] OR “community institutional relation*”[tiab] OR “community participation” [tiab] OR “action research*” [tiab] OR “participatory engag*”[tiab] OR “community research”[tiab] OR “physician scientist*”[tiab] OR “public private partner*”[tiab] OR “regulatory collaborat*”[tiab] OR “industry collaborat*”[tiab] OR community-institutional relations[mesh] OR “community academic partner*”[tiab] OR “community university partner*”[tiab] OR “community academic collaborat*”[tiab] OR “community university collaborat*”[tiab] OR community engag*[ad] OR  “community inform*” OR community-inform*) NOT (letter[pt] OR comment[pt])

**SCOPUS** 
**searched 2/28/2022**

**350 results**

(limited to reviews and articles)

TITLE-ABS-KEY-AUTH ( "interdisciplinary research"  OR  "interdisciplinary science"  OR  "transdisciplinary science"  OR  "transdisciplinary research"  OR  "multidisciplinary science"  OR  "multidisciplinary research"  OR  "transdisciplinary science"  OR  "transdisciplinary research"  OR “interprofessional research” OR “group research” OR “transprofessional research” OR “trans professional research” OR “trans-professional research” OR  ( ( interdisciplinary  OR  inter-disciplinary  OR  transdisciplinary  OR  trans-disciplinary  OR  multidisciplinary  OR  multi-disciplinary  OR  crossdisciplinary  OR  cross-disciplinary )  W/3  ( research*  OR  science  OR  team  OR  teams OR group OR groups ) )  OR  "multisystem science"  OR  "multisystem research"  OR  "multi-system science"  OR  "multi-system research"  OR  "team science"  OR  "team sciences"  OR  scits  OR  "science team"  OR  "science teams"  OR  "team based research"  OR  "team-based research"  OR  "Translational team*" OR “group research” OR “transprofessional research” OR “trans-professional research” )  W/7  ( "Community-Based Participatory Research*"  OR  "community based participatory research*"  OR  "community-based participatory"  OR  cbpr  OR  "community based engagement"  OR  "community based research*"  OR  "community based action research"  OR  "consumer driven research"  OR  "collaborative public health research"  OR  "citizen research"  OR  "participatory research*"  OR  "participatory action research"  OR  "community institutional relation*"  OR  ( ( "community participation"  OR  “community-engag*” OR “community engag*” OR "participatory engag*"  OR  "public private partner*"  OR  "regulatory collaborat*"  OR  "industry collaborat*"  OR  "community academic partner*"  OR  "community university partner*"  OR  "community academic collaborat*"  OR  "community university collaborat*" )  W/5  research* )  OR  "community research"  OR  "physician scientist*" OR “community inform*” OR community-inform* )  AND  ( LIMIT-TO ( DOCTYPE ,  "ar" )  OR  LIMIT-TO ( DOCTYPE ,  "re" ) )

**EMBASE**

**searched 2/28/2022**

**284 results**

('interdisciplinary research'/exp OR 'interdisciplinary research':ti,ab OR 'interdisciplinary science':ti,ab OR 'inter-disciplinary research':ti,ab OR 'inter-disciplinary science':ti,ab OR 'trans-disciplinary team*':ti,ab OR 'multidisciplinary science':ti,ab OR 'multidisciplinary research':ti,ab OR 'multi-disciplinary research*':ti,ab OR 'multi-disciplinary science':ti,ab OR 'cross-disciplinary research*':ti,ab OR 'cross-disciplinary science':ti,ab OR 'cross disciplinary research*' OR 'cross disciplinary science':ti,ab OR 'transdisciplinary science':ti,ab OR 'transdisciplinary research':ti,ab OR 'trans-disciplinary science':ti,ab OR 'trans-disciplinary research':ti,ab OR 'multisystem science':ti,ab OR 'multisystem research':ti,ab OR 'multi-system science':ti,ab OR 'multi-system research':ti,ab OR 'team science':ti,ab OR 'team science':ff OR 'team sciences':ti,ab OR scits OR 'science team':ti,ab OR 'science teams':ti,ab OR 'team research':ti,ab OR 'team based research':ti,ab OR 'team-based research' OR 'translational team*':ti,ab OR 'translational research team*':ti,ab OR 'interprofessional research':ti,ab OR 'group research':ti,ab OR ‘trans-professional research’:ti,ab OR ‘transprofessional research’:ti,ab) AND ('community based participatory research':ti,ab OR 'community-based participatory research':ti,ab OR cbpr:ti,ab OR 'community engag*':ti,ab OR 'community based engagement':ti,ab OR 'community based research*':ti,ab OR 'community based action research*':ti,ab OR 'consumer driven research*':ti,ab OR 'collaborative public health research*':ti,ab OR 'citizen research*':ti,ab OR 'participatory research*':ti,ab OR 'participatory research'/exp OR 'participatory action research':ti,ab OR 'community institutional relation*':ti,ab OR 'community participation':ti,ab OR 'action research*':ti,ab OR 'action research'/exp OR 'participatory engag*':ti,ab OR 'community research':ti,ab OR 'physician scientist*':ti,ab OR 'public private partner*':ti,ab OR 'regulatory collaborat*':ti,ab OR 'industry collaborat*':ti,ab OR 'community-institutional relations'/exp OR 'community academic partner*':ti,ab OR 'community university partner*':ti,ab OR 'community academic collaborat*':ti,ab OR 'community university collaborat*':ti,ab OR 'community engag*':ff OR ‘community inform*’:ti,ab OR community-inform*:ti,ab) AND ('article'/it OR 'article in press'/it OR 'review'/it)

**CINAHL**

**searched 2/28/2022**

**97 results**

(MH "Research, Interdisciplinary" OR TI "interdisciplinary research" OR AB "interdisciplinary research" OR TI "interdisciplinary science" OR AB "interdisciplinary science" OR TI "inter-disciplinary research" OR AB "inter-disciplinary research" OR TI "inter-disciplinary science" OR AB "inter-disciplinary science" OR  TI "transdisciplinary science" OR AB "transdisciplinary science" OR TI "transdisciplinary research" OR AB "transdisciplinary research" OR TI "trans-disciplinary research" OR AB "trans-disciplinary research" OR TI "trans-disciplinary team*" OR AB "trans-disciplinary team*" OR TI "multidisciplinary science" OR AB "multidisciplinary science" OR TI "multidisciplinary research" OR AB "multidisciplinary research" OR TI "multi-disciplinary research*" OR AB "multi-disciplinary research*" OR TI "multi-disciplinary science" OR AB "multi-disciplinary science" OR TI "cross-disciplinary research*" OR AB "cross-disciplinary research*" OR TI "cross-disciplinary science" OR AB "cross-disciplinary science" OR "cross disciplinary research*" OR TI "cross disciplinary science" OR AB "cross disciplinary science" OR TI "transdisciplinary science" OR AB "transdisciplinary science" OR TI "transdisciplinary research" OR AB "transdisciplinary research" OR TI "trans-disciplinary science" OR AB "trans-disciplinary science" OR TI "trans-disciplinary research" OR AB "trans-disciplinary research" OR TI “transprofessional research” OR AB “transprofessional research” OR TI “trans-professional research” OR AB “trans-professional research” OR TI "multisystem science" OR AB "multisystem science" OR TI "multisystem research" OR AB "multisystem research" OR TI "multi-system science" OR AB "multi-system science" OR TI "multi-system research" OR AB "multi-system research" OR TI "team science" OR AB "team science" OR AF "team science" OR TI "team sciences" OR AB "team sciences" OR scits OR TI "science team" OR AB "science team" OR TI "science teams" OR AB "science teams" OR TI "team research" OR AB "team research" OR TI "team based research" OR AB "team based research" OR "team-based research" OR TI "Translational team*" OR AB "Translational team*" OR TI "translational research team*" OR AB "translational research team*" OR TI "interprofessional research" OR AB "interprofessional research" OR TI “group research” OR AB “group research”) AND (TI "community based participatory research" OR AB "community based participatory research" OR TI "community-based participatory research" OR AB "community-based participatory research" OR TI CBPR OR AB CBPR OR TI "community engag*" OR AB "community engag*" OR TI "community based engagement" OR AB "community based engagement" OR TI "community based research*" OR AB "community based research*" OR TI "community based action research*" OR AB "community based action research*" OR TI "consumer driven research*" OR AB "consumer driven research*" OR TI "collaborative public health research*" OR AB "collaborative public health research*" OR TI "citizen research*" OR AB "citizen research*" OR TI "participatory research*" OR AB "participatory research*" OR TI "participatory action research" OR AB "participatory action research" OR TI "community institutional relation*" OR AB "community institutional relation*" OR TI "community participation" OR AB "community participation" OR TI "action research*" OR AB "action research*" OR TI "participatory engag*" OR AB "participatory engag*" OR TI "community research" OR AB "community research" OR TI "physician scientist*" OR AB "physician scientist*" OR TI "public private partner*" OR AB "public private partner*" OR TI "regulatory collaborat*" OR AB "regulatory collaborat*" OR TI "industry collaborat*" OR AB "industry collaborat*" OR TI "community academic partner*" OR AB "community academic partner*" OR TI "community university partner*" OR AB "community university partner*" OR TI "community academic collaborat*" OR AB "community academic collaborat*" OR TI "community university collaborat*" OR AB "community university collaborat*" OR AF "community engag*" OR TI “community inform*” OR TI community-inform***** OR AB “community inform*” OR AB community-inform*****)

**ERIC**

**searched 2/28/2022**

**31 results**

(TI "interdisciplinary research" OR AB "interdisciplinary research" OR TI "interdisciplinary science" OR AB "interdisciplinary science" OR TI "inter-disciplinary research" OR AB "inter-disciplinary research" OR TI "inter-disciplinary science" OR AB "inter-disciplinary science" OR  TI "transdisciplinary science" OR AB "transdisciplinary science" OR TI "transdisciplinary research" OR AB "transdisciplinary research" OR TI "trans-disciplinary research" OR AB "trans-disciplinary research" OR TI "trans-disciplinary team*" OR AB "trans-disciplinary team*" OR TI "multidisciplinary science" OR AB "multidisciplinary science" OR TI "multidisciplinary research" OR AB "multidisciplinary research" OR TI "multi-disciplinary research*" OR AB "multi-disciplinary research*" OR TI "multi-disciplinary science" OR AB "multi-disciplinary science" OR TI "cross-disciplinary research*" OR AB "cross-disciplinary research*" OR TI "cross-disciplinary science" OR AB "cross-disciplinary science" OR "cross disciplinary research*" OR TI "cross disciplinary science" OR AB "cross disciplinary science" OR TI "transdisciplinary science" OR AB "transdisciplinary science" OR TI "transdisciplinary research" OR AB "transdisciplinary research" OR TI "trans-disciplinary science" OR AB "trans-disciplinary science" OR TI "trans-disciplinary research" OR AB "trans-disciplinary research" OR TI "multisystem science" OR AB "multisystem science" OR TI "multisystem research" OR AB "multisystem research" OR TI "multi-system science" OR AB "multi-system science" OR TI "multi-system research" OR AB "multi-system research" OR TI "team science" OR AB "team science" OR AF "team science" OR TI "team sciences" OR AB "team sciences" OR scits OR TI "science team" OR AB "science team" OR TI "science teams" OR AB "science teams" OR TI "team research" OR AB "team research" OR TI "team based research" OR AB "team based research" OR "team-based research" OR TI "Translational team*" OR AB "Translational team*" OR TI "translational research team*" OR AB "translational research team*" OR TI "interprofessional research" OR AB "interprofessional research" OR TI “Transprofessional research” OR AB “Transprofessional research” OR TI “Trans-professional research” OR AB “trans-professional research”) AND (TI "community based participatory research" OR AB "community based participatory research" OR TI "community-based participatory research" OR AB "community-based participatory research" OR TI CBPR OR AB CBPR OR TI "community engag*" OR AB "community engag*" OR TI "community based engagement" OR AB "community based engagement" OR TI "community based research*" OR AB "community based research*" OR TI "community based action research*" OR AB "community based action research*" OR TI "consumer driven research*" OR AB "consumer driven research*" OR TI "collaborative public health research*" OR AB "collaborative public health research*" OR TI "citizen research*" OR AB "citizen research*" OR TI "participatory research*" OR AB "participatory research*" OR TI "participatory action research" OR AB "participatory action research" OR TI "community institutional relation*" OR AB "community institutional relation*" OR TI "community participation" OR AB "community participation" OR TI "action research*" OR AB "action research*" OR TI "participatory engag*" OR AB "participatory engag*" OR TI "community research" OR AB "community research" OR TI "physician scientist*" OR AB "physician scientist*" OR TI "public private partner*" OR AB "public private partner*" OR TI "regulatory collaborat*" OR AB "regulatory collaborat*" OR TI "industry collaborat*" OR AB "industry collaborat*" OR TI "community academic partner*" OR AB "community academic partner*" OR TI "community university partner*" OR AB "community university partner*" OR TI "community academic collaborat*" OR AB "community academic collaborat*" OR TI "community university collaborat*" OR AB "community university collaborat*" OR AF "community engag*" OR TI “community inform*” OR TI community-inform***** OR AB “community inform*” OR AB community-inform*****)
