# Supplementary material for: The intersection of community engagement and team science research: A scoping review
Source: J Clin Transl Sci. 2024 Oct 28;8(1):e208. doi: 10.1017/cts.2024.644 (PMC11713437; doi:10.1017/cts.2024.644)
Supplement: Hohl et al. supplementary material 2 — Hohl et al. supplementary material [file S2059866124006447sup002.pdf]

TS and CE scoping review data abstraction

Please complete the survey below.

Thank you!

Section 1: Study contextual characteristics

Abstractor name

____

Article ID (Use Covidence #)

____

Publication year

____

Full name of journal

____

First author last name (et al. if relevant)

____

Does the manuscript describe author contributions? Check all that apply.

- ☐ Yes, in a separate section as required by journal (I.e., author contributions
- ☐ Yes, in the acknowledgements section.
- ☐ Yes, embedded within manuscript text.
- ☐ No.
- ☐ Other

Enter any text to describe your "other" response to the author contributions question above.

____

Funder(s). Check all that apply.

- ☐ NIH; National Institutes of Health, any agency including NCATS
- ☐ HRSA; Health Resources and Services Administration
- ☐ NSF; National Science Foundation
- ☐ PCORI; Patient Centered Outcomes Research Institute
- ☐ CDC; Centers for Disease Control and Prevention
- ☐ Foundation or private grant
- ☐ Internal/institutional funding
- ☐ No funding (e.g., part of QI or process improvement)
- ☐ Funding not listed
- ☐ Other

You indicated that this project was NIH funded. Please indicate the specific institute that funded this work. Check all that apply.	☐ NCI; National Cancer Institute<br>☐ NCATS; National Center for Advancing Translational Sciences (CTSA funder)<br>☐ NIA; National Institute on Aging<br>☐ NHLBI; National Heart, Lung, and Blood Institute<br>☐ NIDA; National Institute on Drug Abuse<br>☐ NIEHS; National Institute of Environmental Health Sciences<br>☐ NIDDK; National Institute of Diabetes and Digestive and Kidney Diseases<br>☐ NIMHD; National Institute on Minority Health and Health Disparities<br>☐ NIH, unspecified<br>☐ Other NIH agency
Please indicate which NIH agency funded this work	______________________
You indicated that this study was funded by a foundation or private grant. Please specify which foundation or private grant.	______________________
You indicated that this study was funded by internal or institutional funding. Please specify which institution funded this work.	______________________
You indicated that this study was funded by "other". Please indicate the funding source of this study,	______________________
Country/ies in which study took place/ country/ies of origin. Check all that apply.<br><br>If the authors did not state but you are inferring the country of study (i.e. authors are all in the U.S. and the context that they describe sounds like a U.S. hospital) please indicate that you are inferring country from context.	☐ United States<br>☐ Other<br>☐ The authors did not explicitly state the country of origin. I have inferred it from context.
You indicated that this study took place outside of the United States. Please specify country/ies.	______________________

Section 2: Scholarly origin, frameworks, theories, and outcomes

Briefly describe the aims/purpose of the study. In your description, make sure to include terms used by authors.

____

Did this study use theoretical framework(s) or conceptual model(s)? (e.g., social ecological models, theory of planned behavior, four-phased model of transdisciplinary team based research, etc.)

- ◯ Yes
- ◯ No
- ◯ Unsure

List the theoretical framework(s) or conceptual model(s) used in this study. Make sure to use terms used by the authors.

____

Indicate the study population (1). Check all that apply.

- ☐ Humans
- ☐ Non humans

Indicate the HUMAN study population (1a)

- ☐ Individuals with shared experience (e.g., certain health condition)
- ☐ Groups (e.g., research team)
- ☐ Other

Please indicate what other human population was studied in this paper.

____

Indicate the NON-HUMAN study population.

____

In which setting(s) did the study occur? Check all that apply.

- ☐ Schools
- ☐ Centers (i.e., research center or institute)
- ☐ Government program or agency
- ☐ Health center (i.e. clinic, hospital)
- ☐ Other (i.e., communities)

Please indicate in what other setting this study took place.

____

Indicate which methods were used, using, to the extent possible, the language/terms used by authors in the manuscript. Check all that apply.

- ☐ Qualitative interviews
- ☐ Qualitative focus groups
- ☐ Qualitative other, not interviews or focus groups (e.g., observations)
- ☐ Quantitative randomized controlled trial
- ☐ Quantitative non-randomized (i.e., intervention or comparison group)
- ☐ Quantitative descriptive (i.e., single group)
- ☐ Mixed or multi-methods
- ☐ Other

Please indicate what other qualitative methods were used.

____

Please indicate what other methods were used.

____

Please briefly note if methods or approach were difficult to describe and/or do not fit the above categories.

____

What outcomes were reported? (e.g., fruit and veg consumption, screening rates, tobacco cessation, environmental degradation)<br><br>If none, or it was not clearly articulated, please write "none" or "not clearly articulated."<br><br>If authors indicated they would assess or evaluate outcomes, but did not report them, indicate this in the free text section.	__________________________________
Was the partnership itself evaluated?	◯ Yes<br>◯ No
What partnership outcomes were evaluated?	__________________________________

Section 3: Community engagement and team science
Section 3a: Community engagement

In what ways is community engagement showing up? Check all that apply. Specifically, does the manuscript describe:

	Yes	No	Unclear
An academic-community collaboration grounded in reciprocity (i.e. seeking, recognizing, respecting, and incorporating the knowledge, perspectives, and resources that each partner brings to a collaboration)	○	○	○
An asset-based approach, wherein all collaborators' (especially community partners') strengths, skills, and knowledge are respected and incorporated	○	○	○
Co-development of definitions of problems, solutions and/or measures of success	○	○	○
A focus on a community-identified need or priority	○	○	○
Co-design of study (identifying the research question, problem, data collection strategy so the community priority is met)	○	○	○
Community participated in design of data collection instruments	○	○	○
Community participated in study implementation, such as in data collection	○	○	○
Community participated data analysis, interpretation and/or decision making	○	○	○
Community members included as authors on dissemination of findings, e.g., publication, conference presentation, report	○	○	○
The study team reported study findings back to the community.	○	○	○

States policy or practical implications of the research that will benefit the community	○	○	○
Considers sustainability at the outset of the co-design and throughout the research process	○	○	○
Community members are paid members of the study team	○	○	○
Community members are part of advisory boards or councils for the grant or study	○	○	○
Ongoing commitment to community members beyond any single funded project.	○	○	○
Focus on building community capacity beyond the current funding/project focus (i.e., build skills that are transferrable to the next thing/priority)	○	○	○

You indicated that the study described a focus on a community-identified need or priority. Please briefly describe who/what entity in the community identified the need.

____

You indicated that the study described a focus on building community capacity beyond the current funding/project (i.e., build skills that are transferrable to the next thing/priority) . Please briefly describe what capacity was built.

____

Who represented the community in this study? Check all that apply.

- ☐ Community leaders
- ☐ Leaders of specific organizations
- ☐ Members of specific organizations
- ☐ Individuals impacted
- ☐ Other

Please specify who else represented the community that was not captured in the previous question.

____

Please add any comments regarding community engagement in this study that should be noted. You may leave this space blank if you have no comments.

____

Section 3b: Team science

In what ways is team science showing up? Check all that apply. Specifically, does the manuscript describe:

	Yes	No	Unclear
Organizational context of team research (e.g., external support, education, rewards)	○	○	○
Team leadership (i.e., cognitive, motivational, affective, and coordination processes associated with influencing organizational team performance)	○	○	○
Team goals (e.g., purpose of team, goals for teaming)	○	○	○
Roles of team members	○	○	○
How roles are needed to leverage strengths/expertise of people with different roles/from different disciplines and/or sectors (this includes how teams collaborate to achieve breakthroughs unattainable by individual or additive effort)	○	○	○
Team processes developed or utilized to coordinate their work	○	○	○
Interpersonal team effectiveness (how each member feels, behaves, works together)	○	○	○
Facilitating team affect (i.e., Things people are doing to facilitate an effective team dynamic; emotional bonds between team members that are grounded in expressions of genuine care and concern for the welfare of others including empathy, affiliation, and rapport on the basis of shared regard for the others)	○	○	○

Team communication processes/how teams communicate research (i.e., e skill to exchange and integrate knowledge and expertise through interpersonal, relational, organizational, and pedagogical means)	○	○	○
Team research management (i.e., integrating efforts of community and academic partners, e.g., Specific actions taken to organize, plan, and execute components of the research)	○	○	○
Material resources that help team members complete tasks efficiently	○	○	○
Collaborative problem solving (i.e., cognitive and social skills allowing teams to integrate group achievements with team members' idiosyncratic knowledge)	○	○	○

Please add any comments regarding team science in this study that should be noted. You may leave this space blank if you have no comments. ______________________________

Section 3c: Team science and community engagement emphasis

In your assessment, does the manuscript focus more heavily on/emphasize community engagement or team science?

- ○ Community engagement
- ○ Team science
- ○ Equal emphasis on both

What additional comments or questions or questions do you have? ______________________________
