# Supplementary material for: The intersection of community engagement and team science research: A scoping review
Source: J Clin Transl Sci. 2024 Oct 28;8(1):e208. doi: 10.1017/cts.2024.644 (PMC11713437; doi:10.1017/cts.2024.644)
Supplement: Hohl et al. supplementary material 3 — Hohl et al. supplementary material [file S2059866124006447sup003.docx]

## Appendix 3: Funding agencies

| **Funding** |  |  |
| --- | --- | --- |
| Funding external to the institution | 57 | 85.1% |
| Internal or institutional funds | 11 | 16.4% |
| Both internally and externally funded | 6 | 9.0% |
| Multiple funding sources reported | 12 | 17.9% |
| **Funding agency*** |  |  |
| Foundation or private grant | 15 | 22.4% |
| National Institutes of Health, any agency | 13 | 19.4% |
| Centers for Disease Control and Prevention | 2 | 3.0% |
| Patient Centered Outcomes Research Institute | 2 | 3.0% |
| Health Resources and Services Administration | 0 | 0.0% |
| National Science Foundation | 2 | 3.0% |
| Other (e.g., United Kingdom National Health Service, Canadian Institute of Health Research, United States Department of Education) | 34 | 50.7% |
| Internal/institutional funding | 11 | 16.4% |
| Funding not listed | 11 | 16.4% |
| **National Institutes of Health funding agencies represented** | | |
| National Center for Advancing Translational Sciences** | 15 | 22.4% |
| National Heart, Lung, and Blood Institute | 1 | 1.5% |
| National Institute of Allergy and Infectious Disease | 1 | 1.5% |
| National Institute on Drug Abuse | 1 | 1.5% |
| National Institute of Environmental Health Sciences | 3 | 4.5% |
| National Institute on Minority Health and Health Disparities | 1 | 1.5% |
| National Institute of Mental Health | 1 | 1.5% |
| National Institute of Nursing Research | 1 | 1.5% |
| National Institute on Deafness and Other Communication Disorders | 1 | 1.5% |
| NIH, unspecified | 1 | 1.5% |
| NIH Roadmap for Medical Research (NIH Common Fund) | 1 | 1.5% |
| Office of Research on Women's Health | 1 | 1.5% |
| **Private funders***** |  |  |
| ActEarly UK Prevention Research Partnership Consortium |  |  |
| Bill & Melinda Gates Foundation |  |  |
| Carson Foundation |  |  |
| Cogio Foundation |  |  |
| Council on Social Work Education Minority Fellowship Program |  |  |
| Eawag Discretionary Fund (Switzerland) |  |  |
| EU Seventh Framework Programme |  |  |
| Flanders Innovation and Entrepreneurship Agency |  |  |
| Fondo Mixto de Fomento a la Investigación Cientı´fica y Tecnolo´ gica |  |  |
| Health Research Board Partnership Award |  |  |
| Institut Méditerranéen de Recherches Avancées |  |  |
| La Vida Yoga |  |  |
| Leeds South and East Clinical Commissioning group |  |  |
| Netherlands ALS Foundation |  |  |
| Netherlands Organization for Health Research and Development |  |  |
| Rita and Alex Hillman Foundation |  |  |
| Social Science and Humanities Research Grant-Insight Grant |  |  |
| Spenser Foundation |  |  |
| Swedish Research Council for Health, Welfare, and Working Life (FORTE) |  |  |
| Swedish Research Council Formas |  |  |
| Swiss Embassy Guatemala |  |  |
| The Sapelo Foundation |  |  |
| Work for America Foundation |  |  |
| Worldwide Universities Network Fund |  |  |
| *may total more than 100% as some projects had multiple funders | | |
| **Clinical and Translational Science Awards Program funder | | |
